# miR-27a-3p regulates expression of intercellular junctions at the brain endothelium and controls the endothelial barrier permeability

**DOI:** 10.1371/journal.pone.0262152

**Published:** 2022-01-13

**Authors:** Rania Harati, Saba Hammad, Abdelaziz Tlili, Mona Mahfood, Aloïse Mabondzo, Rifat Hamoudi

**Affiliations:** 1 Department of Pharmacy Practice and Pharmacotherapeutics, College of Pharmacy, University of Sharjah, Sharjah, United Arab Emirates; 2 Sharjah Institute for Medical Research, University of Sharjah, Sharjah, United Arab Emirates; 3 Department of Applied Biology, College of Sciences, University of Sharjah, Sharjah, United Arab Emirates; 4 Department of Medicines and Healthcare Technologies, Paris-Saclay University, The French Alternative Energies and Atomic Energy Commission, Gif-sur-Yvette, France; 5 Clinical Sciences Department, College of Medicine, University of Sharjah, Sharjah, United Arab Emirates; 6 Division of Surgery and Interventional Science, University College London, London, United Kingdom; Hungarian Academy of Sciences, HUNGARY

## Abstract

**Background:**

The brain endothelial barrier permeability is governed by tight and adherens junction protein complexes that restrict paracellular permeability at the blood-brain barrier (BBB). Dysfunction of the inter-endothelial junctions has been implicated in neurological disorders such as multiple sclerosis, stroke and Alzheimer’s disease. The molecular mechanisms underlying junctional dysfunction during BBB impairment remain elusive. MicroRNAs (miRNAs) have emerged as versatile regulators of the BBB function under physiological and pathological conditions, and altered levels of BBB-associated microRNAs were demonstrated in a number of brain pathologies including neurodegeneration and neuroinflammatory diseases. Among the altered micro-RNAs, miR-27a-3p was found to be downregulated in a number of neurological diseases characterized by loss of inter-endothelial junctions and disruption of the barrier integrity. However, the relationship between miR-27a-3p and tight and adherens junctions at the brain endothelium remains unexplored. Whether miR-27a-3p is involved in regulation of the junctions at the brain endothelium remains to be determined.

**Methods:**

Using a gain-and-loss of function approach, we modulated levels of miR-27a-3p in an *in-vitro* model of the brain endothelium, key component of the BBB, and examined the resultant effect on the barrier paracellular permeability and on the expression of essential tight and adherens junctions. The mechanisms governing the regulation of junctional proteins by miR-27a-3p were also explored.

**Results:**

Our results showed that miR-27a-3p inhibitor increases the barrier permeability and causes reduction of claudin-5 and occludin, two proteins highly enriched at the tight junction, while miR-27a-3p mimic reduced the paracellular leakage and increased claudin-5 and occludin protein levels. Interestingly, we found that miR-27-3p induces expression of claudin-5 and occludin by downregulating Glycogen Synthase Kinase 3 beta (GSK3ß) and activating Wnt/ß-catenin signaling, a key pathway required for the BBB maintenance.

**Conclusion:**

For the first time, we showed that miR-27a-3p is a positive regulator of key tight junction proteins, claudin-5 and occludin, at the brain endothelium through targeting GSK3ß gene and activating Wnt/ß-catenin signaling. Thus, miR-27a-3p may constitute a novel therapeutic target that could be exploited to prevent BBB dysfunction and preserves its integrity in neurological disorders characterized by impairment of the barrier’s function.

## Introduction

The Blood-Brain Barrier (BBB) is a physiological barrier located at the brain microvasculature and essential for preserving the cerebral homeostasis. The BBB tightly regulates the movement of molecules and cells at the interface between the blood and the brain allowing for a precise control of the brain homeostasis required for proper neuronal function, and a tight protection of the neural tissue from the neurotoxic endogenous and exogenous substances circulating in the blood. Structurally, the BBB is formed by non-fenestrated specialized endothelial cells tightly packed and surrounded by pericytes and perivascular astrocytes. The brain endothelial cells (BEC) possess unique structural, transport and metabolic properties that are essential in creating the barrier. These BECs are joined together by tight and adherence junctional protein complexes that greatly restrict the paracellular permeability. They also express on their luminal and abluminal surface a number of influx and efflux transport proteins regulating the transcellular permeability [1].

Essential in creating a high-resistance paracellular barrier to molecules and ions and in maintaining the barrier integrity, the tight junctions (TJ) are multimolecular complexes located close to the apical membrane. They are composed of integral transmembrane proteins (claudins-1, -3, -5 and -12, occludin and junctional adhesion molecules (JAMs)), major determinants of the TJ permeability, that are connected to the actin cytoskeleton via scaffolding proteins (Zonula-Ocludens ZO1, ZO2, ZO3) linked in turn via cingulin dimers to the actin/myosin cytoskeletal system within the cell. In addition to TJs, junctional complexes between endothelial cells include adherens junctions (AJs) composed of transmembrane proteins (VE-cadherin) linked to the actin cytoskeleton through catenins (including p120-catenin, α- and β-catenin) [2, 3].

Dysfunction of the intercellular junctions at the brain endothelium has been implicated in a number of neuroinflammatory disorders such as multiple sclerosis (MS) [4–7], neurodegenerative disorders such as Alzheimer’s disease [8, 9], ischemic stroke [10, 11], as well as psychiatric disorders including depression and schizophrenia [2, 4, 12]. For instance, in multiple sclerosis, decreased expression or reorganization of junctional proteins including claudin-5, occludin, ZO-1 and VE-cadherin has been associated with loss of BBB integrity and increased transmigration of leukocytes [6, 13, 14]. In Alzheimer’s disease (AD), the amyloid β-peptide was shown to induce changes in TJ and AJ proteins including claudin-5, occludin, ZO-1 and VE-cadherin causing dysfunction of the barrier and increased vascular permeability in AD patients [8, 15, 16]. In stroke and traumatic brain injury, rodent models showed downregulation of junctional proteins including claudin-5 and occludin associated with increased permeability of the barrier and subsequent extravasation of serum proteins [10, 11, 17]. TJ proteins including claudin-5, occludin and ZO-1 were also reported to be downregulated in numerous models of psychiatric disorders [18], depression [19, 20] and schizophrenia [21].

Although the role of junctional dysfunction in the pathogenesis of neurological diseases has been well described in the recent years, our knowledge about the functional regulation of inter-endothelial junctions remains fragmentary. Regulators of inter-endothelial junctions include include Glycogen synthase kinase 3 beta (GSK3ß), Protein kinase C (PKC), Vascular endothelial growth factor (VEGF), Transforming growth factor beta (TGF-β), Matrix metalloproteinases (MMPs) and Tumour Necrosis Factor alpha (TNFα) (reviewed in [4, 22]). However, a better understanding of the regulation of intercellular junctions at the brain endothelium may offer new therapeutic opportunities to preserve or restore the barrier integrity and improve treatment of these disorders.

In recent years, a growing body of evidence showed that brain-enriched microRNAs (miRNAs) act as versatile regulators of the BBB function under physiological and pathological conditions [23, 24]. MicroRNAs are non-coding RNAs, consisting of 21–25 nucleotides that negatively regulate the gene expression at the post-transcriptional level. Altered levels of BBB-associated microRNAs were demonstrated in a number of brain pathologies including neurodegeneration and neuroinflammatory diseases [23–28]. Among the altered micro-RNAs, miR-27a-3p was found to be downregulated in a number of neurological diseases characterized by loss of inter-endothelial junctions and disruption of the barrier integrity. Specifically, miR-27a-3p was found to be downregulated in MS patient’s brain capillaries isolated from active MS lesions compared to capillaries isolated from brains of non-neurological controls or from normal-appearing white matter in MS patients [23]. miR-27a-3p was also found to be significantly reduced in the serum of patients with intracerebral hemorrhage, while intraventricular administration of miR-27a-3p mimic in rats with induced intracerebral hemorrhage attenuated brain edema by targeting aquaporin-11 and protected against the barrier disruption [29]. Reduced levels of miR-27a-3p were also found in the cerebrospinal fluid of patients with Alzheimer’s disease where miR-27a-3p was proposed as a candidate biomarker [30].

Together, these data show a downregulation of miR-27a-3p in neurological diseases characterized by reduction of inter-endothelial junctions expression and disruption of the BBB. However, the relationship between loss of mir-27a-3p and reduction of tight and adherens junctions at the brain endothelium remains unexplored, and whether miR-27a-3p regulates junctions expression at the BBB remains to be determined. In the present study, we investigated the role of miR-27a-3p in the regulation of key TJ (claudin-5, occludin, ZO1) and AJ proteins (VE-cadherin) highly enriched at the brain endothelium *in vitro*. Our results showed that brain endothelial miR-27a-3p induces expression of essential TJ proteins, specifically claudin-5 and occludin, by targeting GSK3ß and activating Wnt/ß-catenin signaling. These findings suggest that miR-27a-3p may represent a potential therapeutic target that could be exploited to restore the BBB integrity in neurological diseases associated with impairment of the barrier’s function.

## Material and methods

### Cell line

The immortalized human cerebral microvascular endothelial cell line (hCMEC/D3) was obtained from Cedarlane (Tebu-Bio, France) and maintained in EndoGRO™-MV Complete Medium (cat# SCME004, EMD Millipore, USA) supplemented with 1ng/mL FGF-2 (cat# GF003, EMD Millipore) and antibiotics at 37°C in 5% CO_2_. Cells were regularly tested for mycoplasma contamination and phenotypic changes.

### Establishment of *in vitro* brain endothelial barriers from hCMEC/D3 cell line

The *in vitro* brain endothelial barrier from hCMEC/D3 cell line was established as previously described [31]. 6 × 10^4^ hCMEC/D3 cells were seeded on the upper apical side of polyester Transwell membranes (Costar, pore size 0.4 μm; growth area 1.12 cm^2^) pre-coated with thin collagen I (cat# 08–115, EMD Millipore) and fibronectin (cat# F1141, Sigma-Aldrich).

### Transient cell transfection

Transfections were performed using the Lipofectamine™ RNAiMAX reagent (Invitrogen) according to the manufacturer’s protocol. To introduce miR-27a-3p, hCMEC/D3 cells were transfected 24 h after culture on transwell membranes with 10 nM of miR-27a-3p mimic (MISSION® microRNA Mimic hsa-miR-27a, Cat# HMI0422, Sigma-Aldrich), or its scrambled control (AllStars Negative Control, cat# SI03650318, Qiagen) and left for 72h. For inhibition studies, 30 nM of miR-27a-3p inhibitor (MISSION® Synthetic microRNA Inhibitor, Human hsa-miR-27a-3p, cat# HSTUD0422, Sigma-Aldrich) or its negative control (miScript Inhibitor Negative Control, cat# 1027271, Qiagen) were transfected using Lipofectamine transfection agent in culture media for 72h. For GSK3ß inhibition, cells were transfected with 20 nM of GSK3B siRNA (cat # sc-35527, Santa Cruz), and for co-transfection of GSK3ß-siRNA/miR-27a-3p miRNA inhibitor, cells were co-transfected with 20 nM of GSK3B siRNA and 30 nM of miR-27a-3p inhibitor or AllStars negative control for 72h. Down- and up-regulation of miR-27a-3p and GSK3ß were assessed by real-time PCR and western blot, respectively.

### Trans-endothelial electric resistance (TEER) measurement

Integrity of the hCMEC/D3 endothelial barriers was monitored 72 h after transfection by measuring the TEER using an Endohm 12 chamber and an Endohmeter EVOMX (World Precision Instruments) as previously described [32]. All TEER values were expressed as Ω.cm2 (surface area of the Transwell inserts) and determined after subtracting the background of the coated transwell membrane and medium from each reading. TEER values of control untransfected endothelial monolayers hCMEC/D3 were higher than 60 Ω.cm^2^ 72h after transfection.

### FITC-dextran paracellular permeability assay

The paracellular permeability of the hCMEC/D3 endothelial barriers was assessed using the FITC-dextran paracellular permeability assay 72 h after transfection as previously described [33, 34]. hCMEC/D3 cells were grown to confluence on transwell polyester membrane inserts (Costar, pore size 0.4 μm; growth area 1.12 cm^2^) pre-coated with collagen I and fibronectin. After 24h, cells were transfected then left for 72 additional hours, and the apical to basolateral paracellular permeability of the hCMEC/D3 monolayers to (4 or 70 kDa) FITC-dextran was investigated. Briefly, 2 mg/ml of (4 or 70 kDa) FITC-dextran were added to the apical compartment in DMEM media without phenol red containing 2% FBS and incubated for one hour at 37°C. After one hour, 100 μl was collected from the apical and the basolateral compartments and the fluorescence of dextran that passed through the endothelial monolayer to the basolateral chamber measured using the BioTek Synergy H1 plate reader at wavelength excitation/emission: 485/528 nm. The volume cleared was plotted against time, and the slopes of the curves used to calculate the permeability coefficients (Pe, cm/s) of the endothelial cell monolayer as previously described [33]: Pe = PS/s where PS (clearance) is the permeability surface area of the endothelial monolayer and s is the surface area of the filter (1.12 cm^2^). PS is calculated as follows: 1/PS = 1/me − 1/mf, where me and mf are the slopes of the curves obtained with the endothelial monolayer on filters and with filters only, respectively. me and mf were calculated by plotting the cleared volume against time. The cleared volume is calculated as follows: (AUa−AUb)/ Fi, where AUa is the total fluorescence (arbitrary units) in the basal compartment, AUb is the background fluorescence and Fi is the fluorescence of the initial solution (AU/ml). PS (clearance) is the permeability surface area of the endothelial monolayer and s is the surface area of the filter (1.12 cm^2^) [33].

### *In silico* bioinformatics analysis

*In silico* bioinformatics analysis was carried out to identify miR-27a-3p target genes that are involved in regulation of the barrier’s properties. We searched for the targets of miR-27a-3p using three prediction databases including TargetScanHuman 7.2, mirTarBase 9.0, and miRDB.org. The target genes identified in the 3 databases were retained.

### Quantitative real-time PCR

For quantitative real-time PCR, total RNAs were extracted from cells using the miRNeasy Micro Kit (cat# 217084, Qiagen, Germany) following the manufacturer’s instructions and as previously described [35]. micro-RNAs were reversed transcribed into cDNA using the miScript II RT Kit (cat# 218161, Qiagen), and mRNAs were reversed transcribed using the RT^2^ First Strand Kit (Cat#, 330401, Qiagen). PCR amplifications were performed on the "Applied Biosystems® StepOne™ Real-Time PCR System" using the miScript SYBR Green PCR Kit (Cat# 218075) for miRNA expression and the RT^2^ SYBR Green ROX qPCR Mastermix (cat# 330522) for mRNA expression according to the manufacturer’s protocol. The primers used were as follows: MystiCq® microRNA qPCR Assay Primer has-miR-27a-3 (cat# MIRAP00068, Sigma Aldrich), RT^2^ qPCR Primer Assay for Human GSK3B (cat# PPH00787C, Qiagen), RT^2^ qPCR Primer Assay for Human CTNNB1 (cat# PPH00643F, Qiagen). Primers of the inter-endothelial junctions were as follows: Claudin-5: forward (5’-ATGTGGCAGGTGACCGCCTTC, Claudin-5 reverse (CGAGTCGTACACTTTGCACTGC); Occludin: forward (5′- TCC ATT GGC AAA GTG AAT GA-3′), Occludin reverse (5′-AGA GGT GCT CTT TTT GAA GG-3′); VE-cadherin forward (5′- GCC AGG TAT GAG ATC GTG GT-3′), VE-cadherin reverse (5′- GTG TCT TCA GGC ACG ACA AA-3′); ZO-1: forward (5′-TGCTGAGTCCTTTGGTGATG-3′), ZO-1 reverse (5′-AATTTGGATCTCCGGGAAGAC-3′). Hs_RNU6-2_11 miScript Primer Assay (cat# MS00033740, Qiagen) and RT^2^ qPCR Primer Assay for Human GAPDH (Cat# PPH72843A, Qiagen) were used as internal control for miRNA and mRNA respectively. The specificity of each reaction was assessed by melting curve analysis and the relative gene expression was determined using the 2^-ΔCt^ and 2^-ΔΔCt^ methods [36].

### Western blot

Protein expression of the inter-endothelial junctions (Claudin-5, Occludin, VE-cadherin, ZO-1), GSK3ß and ß-catenin in hCMEC/D3 cells was assessed by western-blot following standard procedures and as previously described [32, 37, 38]. Cells were lysed on ice with RIPA lysis buffer 1X that contains a cocktail of inhibitors (1 mM PMSF, 1 mM sodium orthovanadate, 1 μg/ml leupeptin, and a protease inhibitor cocktail (cat# P2714, Sigma). Nuclear and membrane proteins were extracted using the NE-PER^TM^ Extraction Reagent ((cat# 78833, Thermofischer) and the Mem-PER^TM^ Plus Membrane Protein Extraction Kit (cat# 89842) respectively and following the manufacturer’s protocol. The following primary antibodies from Abcam were used: Anti-Claudin-5 (ab15106) 1/500, Anti-Occludin (ab216327) 1/1000, VE-cadherin (ab33168) 1/1000, ZO-1 (ab276131) 1/750, Anti-GK3ß ab32391 (1/3500), and Anti-ß-catenin (ab32572) 1/3500. Anti-GAPDH (ab9485) 1/7500 was used as loading and transfer control.

### Dual luciferase reporter assay

The wild-type (WT) putative binding site of miR-27a-3p in the 3′UTR of GSK3B predicted by TargetScan (version 7.2) (position 920–927 of GSK3B 3’ UTR), and the mutant (Mut) 3’UTR of GSK3B with the seed region deleted were cloned into pmirGLO Dual-Luciferase miRNA Target Expression Vector (pmirGLO-empty, Promega, USA) downstream of the firefly luciferase gene (XbaI sites) to obtain Luc Reporter Constructs. The following primers were used for cloning: GSK3B_WT_F: 5’CTAGCTGATGAGCCCAGAGGAAGGGGACAGGTCAGGGATACATCTCACCACTGT GAATAAGTTTGTCCAGATTTTTTTCTAAAGTTA 3’; GSK3B_WT_R: 5’CTAGTAACTTTAGAAAAAAAT CTGGACAAACTTATTCACAGTGGTGAGATGTATCCCTGACCTGTCCCCTTCCTCTGGGCTCATCAG 3’; GSK3B_MUT_F: 5’CTAGCTGATGAGCCCAGAGGAAGGGGACAGGTCATAAGTTTGTCCAGATTTTTTT CTAAAGTTA 3’; GSK3B_MUT_R: 5’CTAGTAACTTTAGAAAAAAATCTGGACAAACTTATGACCTGTCCCCTTCCTCTGGGCTCATCAG 3’. hCMEC/D3 cells were plated at a density of 10^4^ cells/well in a 96-well plate and co-transfected with pmirGLO-Mut (50 ng), pmirGLO-WT (50 ng), miR-27 mimic (10 nM) or negative scrambled control depending on treatments and following Lipofectamine 3000 reagent protocol. 24 h after transfection, cell lysates were harvested and the Firefly and Renilla luciferase activities measured using the Dual-Luciferase® Reporter Assay System analysis (cat# E2940, Promega, USA) according to the manufacturer’s instructions. Firefly luciferase activity was normalized with Renilla luciferase for each sample.

### Statistical analysis

Data are shown as mean ± SD (standard deviation) from three to six independent experiments (biological replicates). Statistical analyses were carried out using independent Student’s t-test for comparison between two groups, one-way ANOVA (analysis of variance) followed by Tukey post hoc test for multiple comparisons between three or four groups, and one-way ANOVA followed by Bonferroni post hoc test for multiple comparisons between five groups. p < 0.05 was considered to be statistically significant and the levels of significance were considered at probability levels of p<0.05(*), p<0.01(**), and p<0.001(***). Calculations and figures were generated using the statistical software GraphPad-Prism (version 8.2.0).

## Results

### Silencing miR-27a-3p increases the brain endothelial barrier permeability and reduces protein expression of claudin-5 and occludin

**Fig 1:**
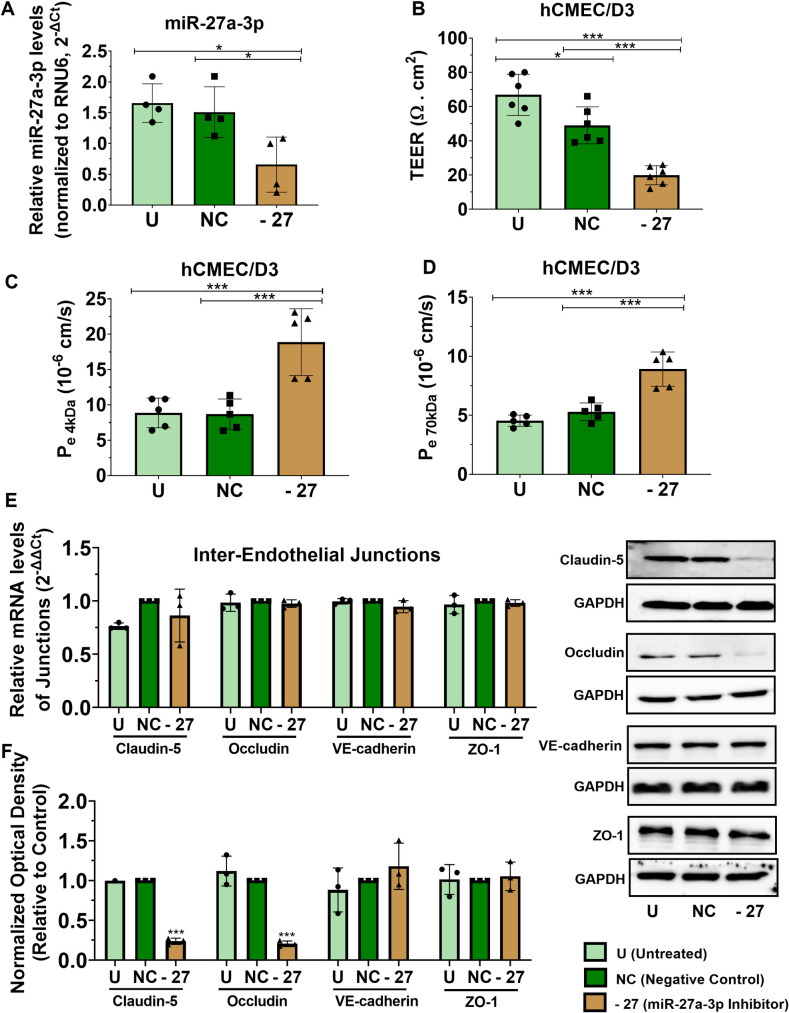
miR-27a-3p inhibitor increases the brain endothelial barrier permeability and downregulates claudin-5 and occludin protein expression. hCMEC/D3 cells were transfected with miR-27a-3p inhibitor or negative control for 72 hours. **(A)** miR-27a-3p relative expression measured by real-time PCR. The small nuclear RNA (RNU6-2) was used as an internal standard. Data are represented as 2^^-ΔCt^. Experiments were carried out four times with PCR performed in duplicates for each experiment. **(B)** Transendothelial electrical resistance (TEER) of hCMEC/D3 cells treated with miR-27a-3p inhibitor or negative control. Experiments were carried out six times with monolayer cultures performed in triplicates. **(C, D)** The permeability coefficient (P_e_, cm/s) of the endothelial monolayer assessed by the 4 (C) and 70 kDa FITX-dextran flux assay (D). Experiments were carried out five times with monolayer cultures performed in triplicates. **(E)** Relative mRNA expression of inter-endothelial junctions (claudin-5, Occludin, VE-cadherin and ZO-1) measured by real-time PCR. GAPDH was used as an internal standard. Data are represented as (2^^-ΔΔCt^). Experiments were carried out three times with PCR performed in duplicates for each experiment. **(F)** Claudin-5, Occludin, VE-cadherin and ZO-1 protein expression measured by western-blot in hCMEC/D3. Optical densities of three independent images were analyzed with Image Lab 6.0.1 software (Bio-Rad) and normalized to GAPDH. Results are represented as normalized optical densities. Experiments were carried out three times with each preparation representing pooled protein lysates from monolayer cultures performed in triplicates. Data represent mean ± SD from the independent experiments (biological replicates). *P<0.*05*, **P<0.*01*, ***P<0.*001*.

**Fig 2:**
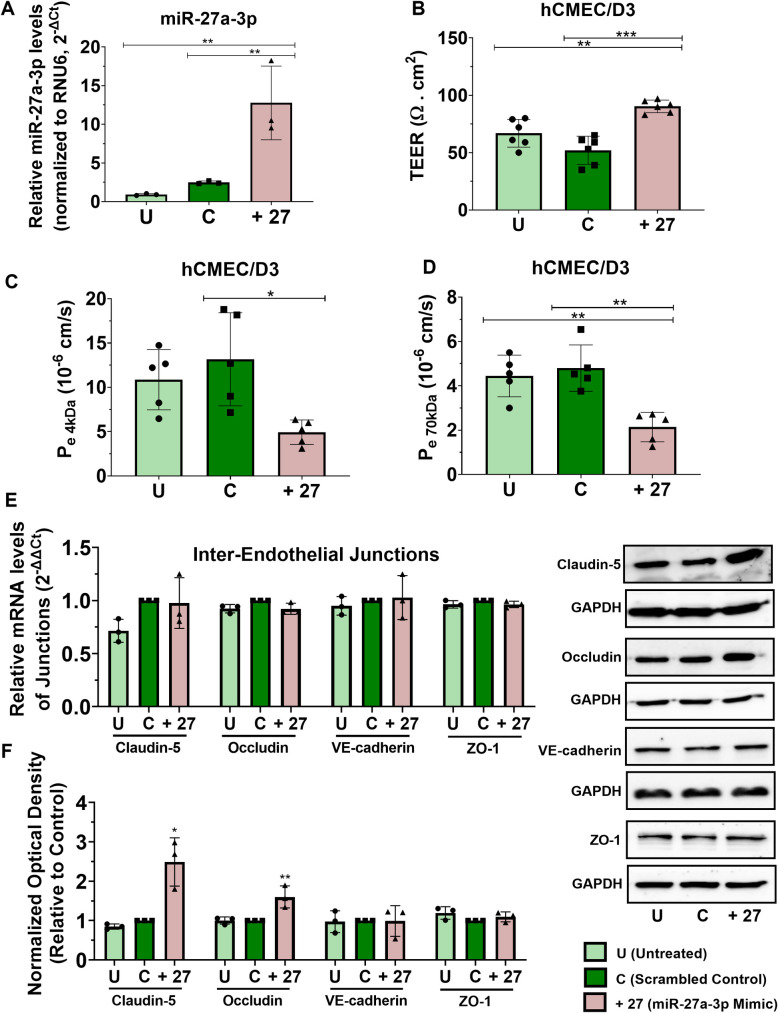
miR-27a-3p mimic reduces the brain endothelial barrier permeability and upregulates claudin-5 and occludin protein expression. hCMEC/D3 cells were transfected with miR-27a-3p mimic or control for 72h. **(A)** miR-27a-3p relative expression measured by real-time PCR. The small nuclear RNA (RNU6-2) was used as an internal standard. Data are represented as 2^^-ΔCt^. Experiments were carried out three times with PCR performed in duplicates for each experiment. **(B)** TEER of hCMEC/D3 cells treated with miR-27a-3p mimic or control. Experiments were carried out six times with monolayer cultures performed in triplicates. **(C, D)** The permeability coefficient (P_e_, cm/s) of the endothelial monolayer assessed by the 4 (C) and 70 kDa FITX-dextran flux assay (D). Experiments were carried out five times with monolayer cultures performed in triplicates. **(E)** Relative mRNA expression of inter-endothelial junctions (claudin-5, Occludin, VE-cadherin and ZO-1) measured by real-time PCR. GAPDH was used as an internal standard. Data are represented as (2^^-ΔΔCt^). Experiments were carried out three times with PCR performed in duplicates for each experiment. **(F)** Claudin-5, Occludin, VE-cadherin and ZO-1 protein expression measured by western-blot in hCMEC/D3. Optical densities of three independent images were analyzed with Image Lab 6.0.1 software(Bio-Rad) and normalized to GAPDH. Results are represented as normalized optical densities. Experiments were carried out three times with each preparation representing pooled protein lysates from monolayer cultures performed in triplicates. Data represent mean ± SD from the independent experiments (biological replicates). *p<0.*05*, **p<0.*01*, ***p<0.*001*.

Downregulation of miR-27a has been reported in a number of neurological diseases characterized by loss of the inter-endothelial tight and adherens junctions and disruption of the barrier integrity [23–25, 27, 29]. The effect of miR-27a-3p on the BBB permeability has been previously investigated, however results are controversial. Indeed, Xi et al. showed that down-regulation of miR-27a-3p is associated with increased BBB permeability, whereas restoration of miR-27a-3p significantly reduced the barrier permeability [29]. By contrast, other studies found that miR-27a inhibitor reduces vascular leakage following ischemic limb injury [39] and in cerebral cavernous malformations [40]. To determine the effect of miR-27a-3p on the brain endothelial permeability, we downregulated miR-27a-3p expression in hCMEC/D3 cells using a mir-27a-3p inhibitor, a small double-stranded RNA that inhibits the mature micro-RNA. hCMEC/D3 cell line is a well-characterized cell line and widely used to study the human BBB *in vitro* as it retains the morphological characteristics of primary brain endothelial cells and expresses a wide range of BBB structural (e.g. tight junctions, cell surface adhesion molecules) and functional components (e.g. the BBB efflux transporters) [33, 41] as well as many micro-RNAs [24]. Effect of miR-27a-3p downregulation on integrity and permeability of the endothelial barrier was examined using the TEER and the dextran permeability assays respectively, while gene and protein expression of essential TJ (claudin-5, occludin, ZO-1) and AJ (VE-cadherin) proteins highly enriched at the brain endothelium and known to be deregulated in multiple neurological disorders was examined using real time PCR and western-blot. hCMEC/D3 transfection with miR-27a-3p inhibitor (30 nM) reduces miR-27a-3p expression when compared to negative control by 58% (p = 0.03) **(Fig 1A)** and this ectopic downregulation remarkably reduced the monolayer’s TEER by 59% (p = 0.00004) **(Fig 2B)** to values around 20 Ω.cm^2^ in cells transfected with miR-27a-3p inhibitor compared to TEER values around 50 Ω.cm^2^ and 70 Ω.cm^2^ in cells transfected with negative control and untreated cells respectively. The paracellular permeability of hCMEC/D3 was examined by measuring the paracellular transport of fluorescently labelled dextrans (FITC-dextran) having low (4 kDa) and high molecular weight (70 kDa). Endothelial cells do not actively transport dextran across compartments and dextran permeability is often used to measure paracellular leakage of the *in-vitro* BBB. Furthermore, hCMEC/D3 cells are known to have a restricted permeability to low and high molecular weight dextrans [42]. FITC-dextran (4 and 70 kDa) were added in the apical compartment and the apical to basolateral (A/B) transport for both molecular weight dextrans was assessed by measuring the paracellular permeability coefficient Pe (cm/s). The A/B paracellular leakage for 4 kDa dextran (apparent permeability coefficient Pe_4kDa_ A/B: 8.7 x 10^−6^ cm/s) was higher than the leakage for 70 kDa dextran (Pe_70kDa_ A/B: 4.9 x 10^-6^cm/s) in untreated cells and in cells transfected with negative control. These values fall within the normal Pe ranges previously described in the hCMEC/D3 monolayers [42, 43]. miR-27a-3p inhibitor significantly increased the paracellular transport of both 4 and 70 kDa dextran by 2.2-fold (p = 0.0009) **(Fig 1C)** and 1.7-fold (p = 0.0002) **(Fig 1D)** respectively. These results show that loss of miR-27a-3p disrupts the brain endothelial barrier integrity as reflected by the reduction in the TEER value, and increases the paracellular leakage of low and high molecular weight dextrans through the inter-endothelial junctions. These findings are in accordance with previous studies showing that down-regulation of miR-27a-3p increases the permeability of brain endothelial cells to dextran 20 and impairs the proliferation of BECs [29]. We next assessed the gene and protein expression of the inter-endothelial junctions claudin-5, occludin, ZO-1 and VE-cadherin following downregulation of miR-27a-3p. Our results show that miR-27a-3p inhibitor does not significantly affect the gene expression of these junctions **(Fig 1E**). However, miR-27a-3p inhibitor causes a significant downregulation of claudin-5 protein expression by 80% (p = 0.0003) compared to cells transfected with negative control **(Fig 1F)** which explains the increase in 4kDa FITC-dextran permeability. Indeed, claudin-5 is known to restrict paracellular permeability to low molecular weight molecules, where loss of claudin-5 was shown to increase paracellular permeability to small molecules [5]. In addition, miR-27a-3p inhibitor significantly reduces occludin protein expression by 76% (p<0.0001), explaining the increased leakage of 70 kDa FITC-dextran [44]. Analysis of the effect of miR-27a-3p inhibitor on the TJ protein ZO-1 and the AJ protein VE-cadherin showed no significant effect on the protein levels of these junctions. Taken together, our results showed that miR-27a-3p inhibitor increases the brain endothelial barrier permeability by downregulating protein expression of the TJ proteins claudin-5 and occludin, with no effect on ZO-1 and VE-cadherin levels. However, this does not exclude that the increased barrier permeability could also be due to complex regulatory mechanisms induced by loss of miR-27a-3p at the brain endothelium and affecting other inter-endothelial junctions as well as BECs proliferation [29].

### miR-27a-3p mimic reduces the brain endothelial permeability and increases claudin-5 and occludin protein expression

We next assessed whether the ectopic increase of miR-27a-3p in the brain endothelial cells enhances the barrier integrity, reduces the paracellular permeability and increases expression of the inter-endothelial junctions. hCMEC/D3 cells were transfected with miR-27a-3p mimic (10 nM) or scrambled control and the resultant effects on the monolayer’s TEER, dextrans paracellular permeability and gene and protein expression of the inter-endothelial junctions (claudin-5, occludin, ZO-1, VE-cadherin) were examined. Our results represented in Fig 2 show that transfection of hCMEC/D3 cells with mir-27a-3p mimic induces expression of the micro-RNA (fold change = 5.1, p = 0.009) **(Fig 2A)**, and this increase was associated with increase in the TEER values (fold change = 1.74-fold, p<0.0001) **(Fig 2B)** and a reduction in the permeability to 4kDa FITC-dextran (fold change = 0.38, p = 0.01) **(Fig 2C)** and to 70kDa FITC-dextran (fold change = 0.45, p = 0.0014) **(Fig 2D)** compared to control. Interestingly, miR-27a-3p was also associated with an increase in the protein expression of claudin-5 (fold change = 1.6, p = 0.01) and occludin (fold change = 2.5, p = 0.005) compared to the negative control with no changes in their gene expression, nor in the gene and protein expression of ZO-1 and VE-cadherin **(Fig 2E and 2F)**.

### miR-27a-3p regulates claudin-5 and occludin protein expression by targeting GSK3ß and activating Wnt/ß-catenin signaling

**Fig 3:**
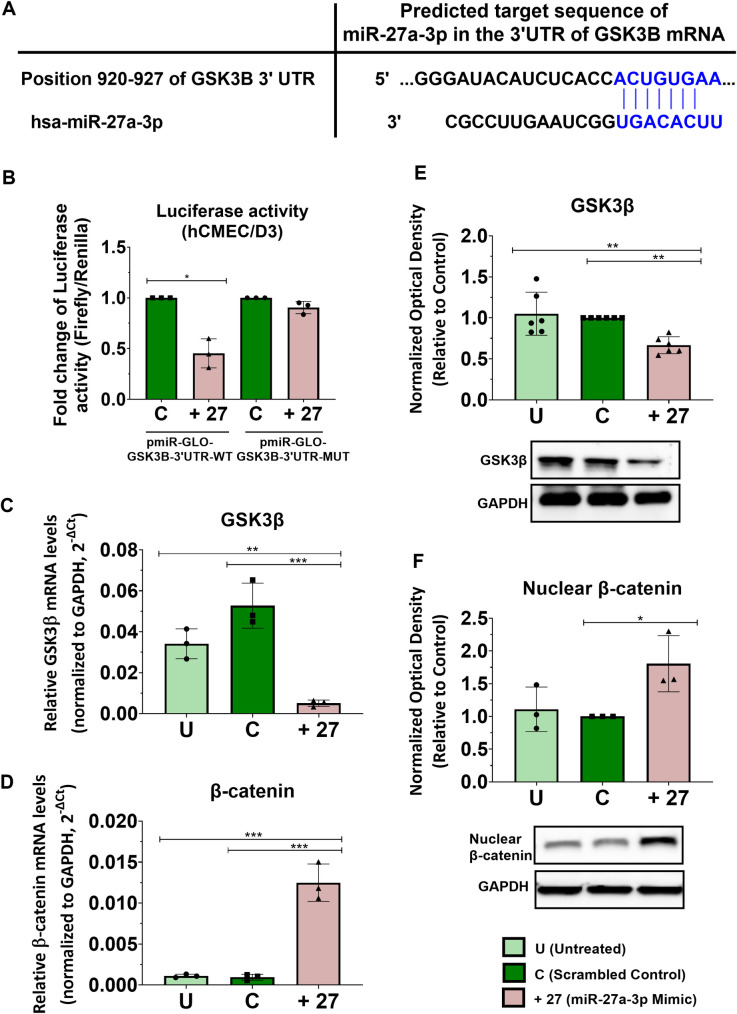
miR-27a-3p regulates claudin-5 and occludin expression by targeting GSK3ß and upregulating Wnt/ß-catenin. **(A)** Schematic representation of the GSK3ß 3’-UTR with the miR-27a-3p binding site. Complementary sequences are represented in blue. **(B)** Effect of miR-27a-3p mimic on GSK3ß 3’UTR luciferase reporters (wild type and mutant). Results are expressed as the fold change of the ratio (firefly to Renilla luciferase activity). Experiments were carried out three times with tests performed in triplicates for each experiment. **(C, D)** Relative mRNA expression of GSK3ß (C) and ß-catenin (D) measured by real-time PCR in hCMEC/D3 cells transfected with miR-27a-3p mimic or control for 72h. GAPDH was used as an internal standard. Data are represented as (2^^-ΔCt^). Experiments were carried out three times with PCR performed in duplicates for each experiment. **(E, F)** Protein expression of the GSK3ß (E) and nuclear ß-catenin (F) measured by western-blot in hCMEC/D3. Optical densities of three independent images were analyzed with Image Lab 6.0.1 software(Bio-Rad) and normalized to GAPDH. Results are represented as normalized optical densities. Experiments were carried out three to six times with each preparation representing pooled protein lysates from monolayer cultures performed in triplicates. Data represent mean ± SD from the independent experiments (biological replicates). *p<0.*05*, **p<0.*01*, ***p<0.*001*.

**Fig 4:**
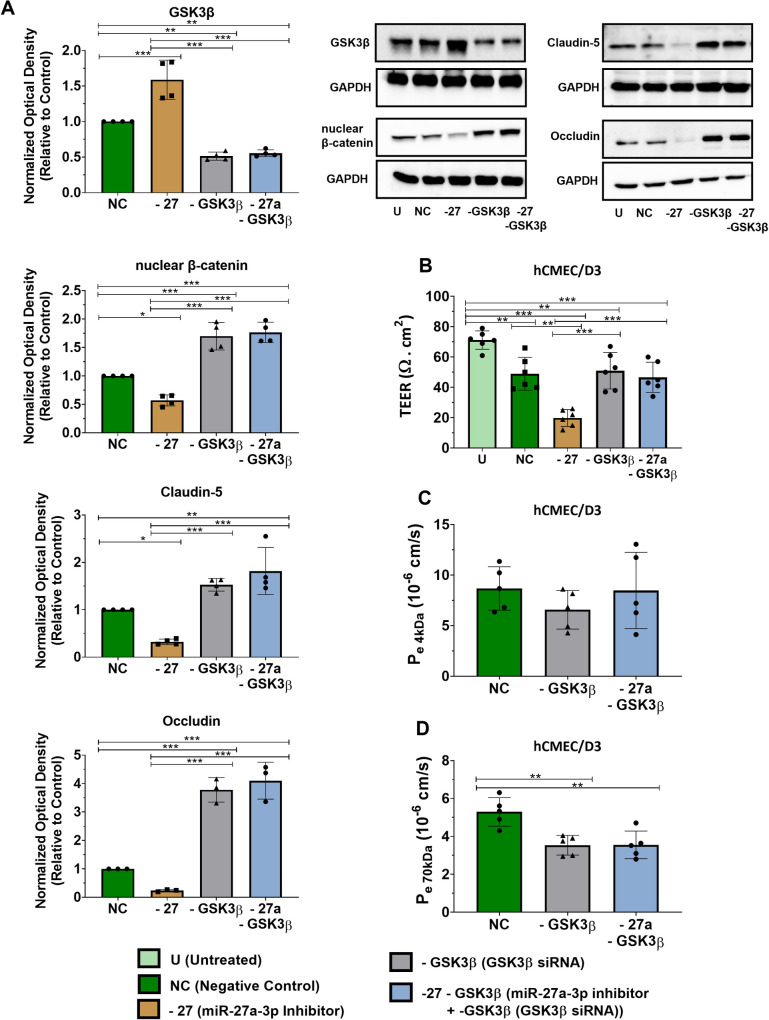
GSK3ß inhibition rescues the activation of GSK3ß by miR-27a-3p inhibitor. hCMEC/D3 cells were transfected with miR-27a-3p inhibitor and/or GSK3B siRNA or negative control for 72h. **(A)** Protein expression of GSK3ß, nuclear ß-catenin, claudin-5 and occludin measured by western-blot in hCMEC/D3. Optical densities of three independent images were analyzed with Image Lab 6.0.1 software(Bio-Rad) and normalized to GAPDH. Results are represented as normalized optical densities. Experiments were carried out three to four times with each preparation representing pooled protein lysates from monolayer cultures performed in triplicates. **(B)** Transendothelial electrical resistance (TEER) of hCMEC/D3 cells treated with miR-27a-3p inhibitor and/or GSK3B siRNA or negative control after 72 hours of transfection. Experiments were carried out six times with monolayer cultures performed in triplicates. **(C, D)** The permeability coefficient (P_e_, cm/s) of the endothelial monolayer assessed by the 4 (C) and 70 kDa FITX-dextran flux assay (D). Experiments were carried out five times with monolayer cultures performed in triplicates. Data represent mean ± SD from the independent experiments (biological replicates). *p<0.*05*, **p<0.*01*, ***p<0.*001*.

Our results showed that miR-27a-3p regulates the barrier permeability, where miR-27a-3p inhibitor is associated with loss of claudin-5 and occludin expression, while miR-27a-3p mimic causes a significant increase in claudin-5 and occludin protein expression. We next investigated the mechanism by which miR-27a-3p regulates claudin-5 and occludin expression and the barrier permeability. To identify miR-27a-3p target gene that regulate the barrier’s properties, we carried out an *in silico* bioinformatics analysis where we searched for miR-27a-3p targets using three prediction databases: TargetScanHuman 7.2, mirTarBase 9.0, and miRDB.org. Interestingly, all three databases identified GSK3ß (glycogen synthase kinase-3 beta) as miR-27a-3p target. GSK3B was also confirmed experimentally as validated target of miR-27a-3p by previous studies where miR-27a-3p was confirmed to target the 3’UTR region of GSK3B mRNA and reduces its expression using the Dual-Glo luciferase reporter assay, which activates the Wnt/ß-catenin pathway [45, 46]. Wnt/ß-catenin is a key signaling pathway regulating the BBB development and maintenance under various pathophysiological conditions. We and others have shown that activation of the Wnt/β-catenin pathway promotes the formation of tight junctions and induces transport proteins in the BBB during brain development and maturation [38, 47–49]. Dysregulation of Wnt/β-catenin pathways has been implicated in neurological disorders characterized by BBB dysfunction including multiple sclerosis [50], stroke [51] and Alzheimer’s disease [52]. GSK3ß is a serine/threonine protein kinase that inhibits Wnt/ß-catenin signaling by inducing β-catenin phosphorylation targeting thereby the protein for ubiquitination and subsequent proteosomal degradation. Inhibition of GSK3ß stabilizes β-catenin which allows its translocation to the nucleus and induction of gene transcription [53, 54]. Activation of Wnt signaling through GSK3ß inhibition was shown to improve the BBB phenotype in cultured brain endothelial hCMEC/D3, resulting in reduced paracellular permeability [55], and treatment of hCMEC/D3 cells with GSK3β inhibitor increases barrier properties and promotes claudin-5 and occludin protein stability [56]. In addition, in aged mice, claudin-5 protein expression was found to be reduced in brain endothelial cells, and the inhibition of GSK3ß was shown to upregulate claudin-5 at the BBB, reduce the barrier permeability, restore its integrity, and improve cognitive functions [57]. To examine whether miR-27a-3p regulates claudin-5 and occludin through inhibition of GSK3ß and activation of Wnt/ß–catenin signaling, we first confirmed previous observations reporting GSK3ß as a target of miR-27a-3p using luciferase reporter assay in hCMEC/D3 cells. The putative target sequence of miR-27a-3p in the 3’UTR of GSK3ß mRNA predicted by TargetScan 7.2 is shown in **Fig 3A**. miR-27a-3p mimic was co-transfected with luciferase constructs containing the putative (wild-type WT) or mutated (Mut) binding site of miR-27a-3p (pmirGlo-GSK3B (seed-miR-27)-3’UTR) in the 3’UTR of GSK3ß. The luciferase assays showed that miR-27a-3p mimic significantly reduced the luciferase activity in cells transfected with the pmirGlo-GSK3ß-3’UTR-WT construct compared to control cells by 54% (p = 0.02), whereas the mimics had no inhibitory effect on luciferase activity in cells transfected with construct containing mutated seed sequences **(Fig 3B)**. These data confirmed previous results showing that miR-27a-3p directly targets GSK3ß and suggest that the effect of miR-27a-3p on claudin-5 and occludin protein expression may be mediated by the downregulation of GSK3ß. We next investigated the effect of miR-27p-3p mimic on gene and protein expression of GSK3ß. hCMEC/D3 cells were transfected with miR-7a-3p mimic (10 nM) or scrambled control and gene and protein expression of GSK3ß was examined by real-time PCR and western blot respectively. Results show that treatment with miR-27a-3p mimic significantly reduced GSK3ß gene and protein expression (p<0.05) **(Fig 3C and 3E)**. Inhibition of GSK3ß was associated with activation of ß-catenin as evidenced by the increase of the gene expression and the nuclear proteins levels of ß-catenin (p<0.05) **(Fig 3D and 3F)**. To confirm the effect of miR-27a-3p on GSK3ß, hCMEC/D3 cells were co-transfected with miR-27a-3p inhibitor and/or GSK3ß siRNA. Our results showed that the co-transfection caused GSK3ß knockdown preventing its upregulation by miR-27 inhibitor, while this co-transfection caused upregulation of nuclear ß-catenin, claudin-5 and occludin and prevented their downregulation by miR-27a-3p inhibition **(Fig 4A)**. We also examined levels of ß-catenin in the membrane fraction. Indeed, in the brain endothelial cells, β-catenin has a double role, where it has both a transcription co-transactivator role and a structural role as part of the adherens junctions. As a co-transactivator, the nuclear β-catenin associates with the lymphoid enhancer factor (LEF)/T-cell factor (TCF) family of transcription factors and mediates expression of target genes. As a structural protein, the membrane pools of β-catenin interacts with cytoskeletal proteins and members of adherens complexes to promote stability of the intercelllular junctions [58]. Thus, membrane levels of β-catenin are involved in the stability of the junctional proteins. Western blot performed on the membrane fractions of ß-catenin showed a reduction in protein levels following miR-27a-3p inhibition, however co-transfection of hCMEC/D3 cells with miR-27a-3p inhibitor and/or GSK3ß siRNA caused upregulation of the membrane pools of ß-catenin and prevented their downregulation by miR-27a-3p inhibitor **(S1A Fig)**. Interestingly, transfection of miR-27a-3p mimic induced expression of ß-catenin in the membrane fraction (fold change = 2.2, p = 0.0015) **(S1B Fig)**. These results indicate that miR-27a-3p also affects levels of the structural β-catenin at the membrane, where miR-27a-3p induces accumulation of β-catenin which may redistribute to the junction in brain endothelial cells, thereby promoting stability of the junctional complexes.

We also verified whether any of the inter-endothelial junctions is a putative target of miR-27a-3p. *In silico* analysis of the putative target genes using the three prediction database (TargetScanHuman 7.2, mirTarBase 9.0, and miRDB.org) showed that claudin-5, occludin and ZO-1 as well as other junction associated proteins such as LSR1 (lipolysis-stimulated lipoprotein receptor) are not putative targets of miR-27a-3p. However, the *in silico* analysis showed that VE-cadherin is a predicted target of miR-27a-3p. Our experimental analysis showed no effect of miR-27a-3p on the gene and protein expression of VE-cadherin. This could be due to the complex and intersecting regulatory pathways that regulate expression of the inter-endothelial junctions at the brain endothelium which can provide opportunities for feedbacks and interactions involving multiple signals and signaling pathways [22].

Finally, we evaluated the effect of the different transfections on the BBB integrity and paracellular permeability. Our results showed that co-transfecting hCMEC/D3 with miR-27a-3p inhibitor and GSK3ß siRNA increases the endothelial barrier TEER **(Fig 4B)** and reduces its paracellular permeability to 4kDa and 70kDa FITC-dextrans rescuing therefore the effects of miR-27a-3p inhibitor **(Fig 4C and 4D)**. Taken together, our data show that miR-27a-3p regulates expression of the inter-cellular junctions claudin-5 and occludin and the endothelial barrier permeability by downregulating GSK3ß and activating Wnt/ß -catenin signaling.

## Discussion

Downregulation of the brain endothelial miR-27a-3p has been demonstrated in several neurologic disorders characterized by loss of inter-endothelial junctions and disruption of the BBB integrity [4, 10, 15, 23, 24, 29, 30]. In this study, we provide evidence that brain endothelial miR-27a-3p regulates key tight junction proteins, claudin-5 and occludin, by targeting GSK3ß and activating Wnt/ß-catenin signaling. We propose miR-27a-3p as a therapeutic target that can be exploited to restore or prevent BBB dysfunction in neurological disorders.

Using an *in-vitro* model of the brain endothelium, we showed that miR-27a-3p inhibitor reduces claudin-5 and occludin protein expression and increases the barrier paracellular permeability while miR-27a-3p mimic enhances the trans-endothelial electrical resistance and reduces the barrier permeability by upregulating claudin-5 and occludin. This observation is in line with previous studies showing that miR-27a-3p down-regulation in brain endothelial cells (BECs) increases the barrier permeability and impaired proliferation and migration of BECs *in vitro*, while intraventricular administration of miR-27a-3p mimic protects against BBB disruption in rats with induced intracerebral hemorrhage [29]. In contrast, other studies found that miR-27a mimic increases the barrier permeability while miR-27a-3p downregulation reduces vascular leakage following ischemic limb injury [39] and in cerebral cavernous malformations [40]. However, miR-27a-3p was found to be significantly reduced in several brain diseases characterized by loss of claudin-5 and increased barrier permeability, including multiple sclerosis [7, 23, 24], intracerebral hemorrhage [29], Alzheimer’s diseases [30], Huntington’s disease [59] and traumatic brain injury [60], while miR-27a-3p elevation was shown to exert neuroprotective effects. Our results suggest that loss of endothelial miR-27a-3p could be a critical event contributing to loss of claudin-5 and occludin, and therefore barrier dysfunction in neurological diseases, and restoring its expression could be a potential therapeutic strategy to preserve the barrier integrity.

We found that miR-27a-3p affects the barrier permeability by regulating protein expression of claudin-5 and occludin through GSK3ß-mediated activation of Wnt/ß-catenin signaling. Wnt/ß-catenin is a key signaling pathways regulating the BBB development and maintenance under various pathophysiological conditions, and its dysfunction has been reported in a number of neurological diseases characterized by reduced levels of claudin-5 and occludin, and increased vascular permeability [48–52]. For instance, dysfunctional Wnt/β-catenin signaling was shown to contributes to BBB breakdown in Alzheimer’s disease [52]. In addition, BBB dysfunction secondary to defective β-catenin transcription activity was shown to be a key pathogenic factor in hemorrhagic stroke, seizure activity, and CNS inflammation [51]. GSK3ß is a serine/threonine protein kinase that inhibits Wnt/ß-catenin signaling by inducing β-catenin phosphorylation targeting thereby the protein for ubiquitination and subsequent proteosomal degradation. Inhibition of GSK3ß stabilizes β-catenin which allows its translocation to the nucleus and induction of gene transcription [53, 54]. Extensive studies in recent years have shown that dysregulation of GSK3β is a key event that contributes to the development and progression of several neurological disorders, and inhibitors of GSK3β have been shown to be beneficial in many neuroinflammatory disease models including Alzheimer’s disease, multiple sclerosis and AIDS dementia complex (reviewed in [61]. At the BBB, activation of Wnt signaling through GSK3ß inhibition was shown to improve the barrier phenotype in cultured brain endothelial cells hCMEC/D3, resulting in reduced paracellular permeability and enhanced tight junctions protein stability [55, 56]. In disease context, GSK3ß was found to be upregulated in ischemic brain injury and inhibition of GSK3ß protects against BBB disruption [62]. Our results show that GSK3ß is a direct target of miR-27a-3p where miR-27a-3p mimic inhibits GSK3ß causing stabilization of the nuclear ß-catenin and upregulation of claudin-5 and occludin protein expression. These findings suggest that loss of endothelial miR-27a-3p may be a critical event causing upregulation of GSK3ß, downregulation of Wnt/ß-catenin and dysfunction of the inter-endothelial junctions, claudin-5 and occludin, at the brain endothelial barrier in neurological disorders. Further *in vivo* studies are needed to confirm these *in vitro* findings. Effect of miR-27a-3p on other inter-endothelial junctions merits also to be exploited by subsequent studies.

## Conclusion

The present *in vitro* study sheds light on the axis miR-27a-3p/GSK3ß/ß-catenin as a potential therapeutic target that could be exploited by subsequent *in vivo* and translational studies to prevent BBB dysfunction and preserves its integrity in neurological disorders characterized by impairment of the BBB function.

## Supporting information

S1 Fig Effect of miR-27a-3p on β-catenin expression in the membrane fraction of hCMEC/D3 cells. **(A, B)** hCMEC/D3 cells were transfected with miR-27a-3p inhibitor and/or GSK3B siRNA or negative control for 72h (A). hCMEC/D3 cells were transfected with miR-27a-3p mimic or control for 72h (B). Membrane proteins were extracted and protein expression of ß-catenin in the membrane fraction was measured by western-blot in hCMEC/D3. Optical densities of three independent images were analyzed with Image Lab 6.0.1 software(Bio-Rad) and normalized to GAPDH. Results are represented as normalized optical densities. Experiments were carried out three times with each preparation representing pooled protein lysates from monolayer cultures performed in triplicates. Data represent mean ± SD from the independent experiments (biological replicates). *p<0.*05*, **p<0.*01*, ***p<0.*001*.
(TIF)

S1 File (DOCX)

S1 Raw images (DOCX)
