# Supplementary figures and images for: miR-27a-3p regulates expression of intercellular junctions at the brain endothelium and controls the endothelial barrier permeability
Source: PLoS One. 2022 Jan 13;17(1):e0262152. doi: 10.1371/journal.pone.0262152 (PMC8758013; doi:10.1371/journal.pone.0262152)

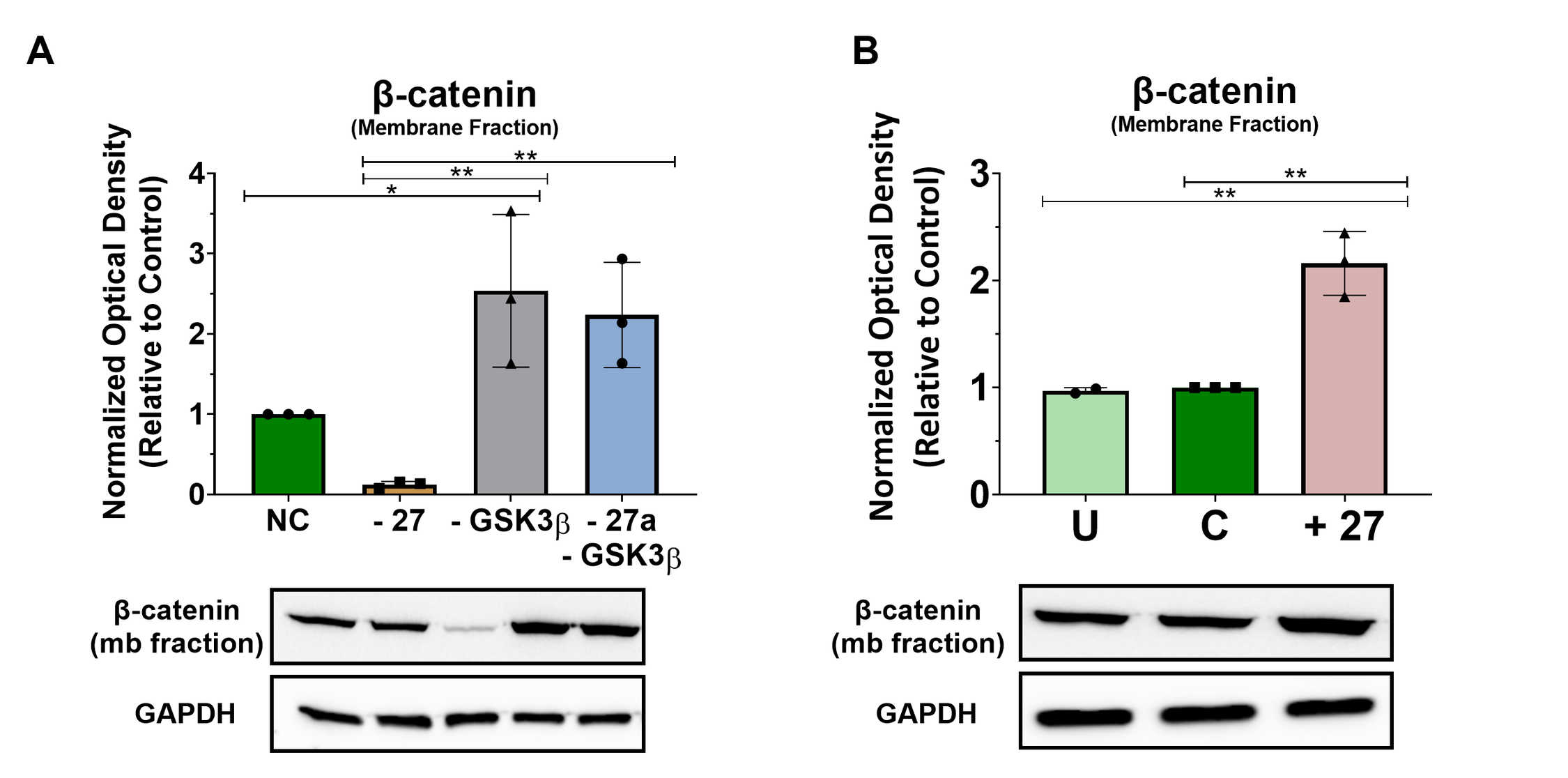

Supplement: S1 Fig — (A, B) hCMEC/D3 cells were transfected with miR-27a-3p inhibitor and/or GSK3B siRNA or negative control for 72h (A). hCMEC/D3 cells were transfected with miR-27a-3p mimic or control for 72h (B). Membrane proteins were extracted and protein expression of ß-catenin in the membrane fraction was measured by western-blot in hCMEC/D3. Optical densities of three independent images were analyzed with Image Lab 6.0.1 software(Bio-Rad) and normalized to GAPDH. Results are represented as normalized optical densities. Experiments were carried out three times with each preparation representing pooled protein lysates from monolayer cultures performed in triplicates. Data represent mean ± SD from the independent experiments (biological replicates). *p<0.05, **p<0.01, ***p<0.001. (TIF) [file pone.0262152.s001.tif]
