# Supplementary material for: miR-27a-3p regulates expression of intercellular junctions at the brain endothelium and controls the endothelial barrier permeability
Source: PLoS One. 2022 Jan 13;17(1):e0262152. doi: 10.1371/journal.pone.0262152 (PMC8758013; doi:10.1371/journal.pone.0262152)
Supplement: S1 File — (DOCX) [file pone.0262152.s002.docx]

**miR-27a-3p regulates expression of intercellular junctions at the brain endothelium and controls the endothelial barrier permeability**

**Short Title: Regulation of inter-endothelial junctions by miR-27a-3p**

**Rania Harati^1,2*^, Saba Hammad^1,2^, Abdelaziz Tlili^3^, Mona Mahfood^3^, Aloïse Mabondzo^4^ & Rifat Hamoudi^2,5,6^**

^1^Department of Pharmacy Practice and Pharmacotherapeutics, College of Pharmacy, University of Sharjah, 27272, Sharjah, United Arab Emirates

^2^Sharjah Institute for Medical Research, University of Sharjah, 27272, Sharjah, United Arab Emirates

^3^Department of Applied Biology, College of Sciences, University of Sharjah, 27272, Sharjah, United Arab Emirates

^4^Paris-Saclay University, Department of Medicines and Healthcare Technologies, The French Alternative Energies and Atomic Energy Commission, 91191, Gif-sur-Yvette, France

^5^Clinical Sciences Department, College of Medicine, University of Sharjah, 27272, Sharjah, United Arab Emirates

^6^Division of Surgery and Interventional Science, University College London, W1W 7EJ London, United Kingdom

Corresponding author: Rania Harati; [rharati@sharjah.ac.ae](mailto:rharati@sharjah.ac.ae)

**Minimal Data Set**

**Fig 1**

| **Figure Number** | **Number of Experiments** | **Individual values** | **Mean** | **S.D.** | **Statistical method used** | ***p* value** |
| --- | --- | --- | --- | --- | --- | --- |
| **Fig 1A: Relative miR-27a-3p levels measured by PCR (2^-ΔCt^)** | | | | | | |
| Untreated (U) | 4 independent experiments (biological replicates), with PCR performed in duplicates (technical replicates) for each experiment | 1.628819 | 1.655654 | 0.313186 | One-way ANOVA followed by Tukey post hoc test | U vs. NC: 0.8604 U vs. - 27: 0.0148 NC vs. - 27: 0.0334 |
|  |  | 1.558199 |  |  |  |  |
|  |  | 1.346103 |  |  |  |  |
|  |  | 2.089493 |  |  |  |  |
| Negative Control (NC) |  | 1.421439 | 1.508653 | 0.410743 |  |  |
|  |  | 2.089493 |  |  |  |  |
|  |  | 1.402044 |  |  |  |  |
|  |  | 1.121637 |  |  |  |  |
| miR-27a-3p Inhibitor (- 27) |  | 0.998693 | 0.657510 | 0.447697 |  |  |
|  |  | 1.083608 |  |  |  |  |
|  |  | 0.206895 |  |  |  |  |
|  |  | 0.340844 |  |  |  |  |
| **Fig 1B: TEER (Ω.cm^2^)** | | | | | | |
| Untreated (U) | 6 independent experiments (biological replicates), with monolayer cultures performed in triplicates (technical replicates) | 72 | 66.833333 | 12.05681 | One-way ANOVA followed by Tukey post hoc test | U vs. NC: 0.018 U vs. - 27: <0.0001 NC vs. - 27: 0.0004 |
|  |  | 79 |  |  |  |  |
|  |  | 61 |  |  |  |  |
|  |  | 80 |  |  |  |  |
|  |  | 59 |  |  |  |  |
|  |  | 50 |  |  |  |  |
| Negative Control (NC) |  | 57 | 49.000000 | 10.807405 |  |  |
|  |  | 66 |  |  |  |  |
|  |  | 42 |  |  |  |  |
|  |  | 39 |  |  |  |  |
|  |  | 40 |  |  |  |  |
|  |  | 50 |  |  |  |  |
| miR-27a-3p Inhibitor (- 27) |  | 26 | 19.833333 | 5.5647701 |  |  |
|  |  | 19 |  |  |  |  |
|  |  | 15 |  |  |  |  |
|  |  | 25 |  |  |  |  |
|  |  | 12 |  |  |  |  |
|  |  | 22 |  |  |  |  |
| **Fig 1C: Pe_4kDa_ A/B (10^-6^ cm/s)** | | | | | | |
| Untreated (U) | 5 independent experiments (biological replicates), with monolayer cultures performed in triplicates (technical replicates) | 6.98 | 8.868 | 2.0957743 | One-way ANOVA followed by Tukey post hoc test | U vs. NC: 0.9955 U vs. - 27: 0.001 NC vs. - 27: 0.0009 |
|  |  | 10.82 |  |  |  |  |
|  |  | 9.32 |  |  |  |  |
|  |  | 6.39 |  |  |  |  |
|  |  | 10.83 |  |  |  |  |
| Negative Control (NC) | 5 independent experiments (biological replicates), with monolayer cultures performed in triplicates (technical replicates) | 6.81 | 8.682 | 2.1480968 |  |  |
|  |  | 11.35 |  |  |  |  |
|  |  | 8.67 |  |  |  |  |
|  |  | 6.35 |  |  |  |  |
|  |  | 10.23 |  |  |  |  |
| miR-27a-3p Inhibitor (- 27) | 5 independent experiments (biological replicates), with monolayer cultures performed in triplicates (technical replicates) | 13.73 | 18.87 | 4.727621 |  |  |
|  |  | 23.1 |  |  |  |  |
|  |  | 21.58 |  |  |  |  |
|  |  | 13.72 |  |  |  |  |
|  |  | 22.22 |  |  |  |  |
| **Fig 1D: Pe_70kDa_ A/B (10^-6^ cm/s)** | | | | | | |
| Untreated (U) | 5 independent experiments (biological replicates), with monolayer cultures performed in triplicates (technical replicates) | 3.9 | 4.54 | 0.4767075 | One-way ANOVA followed by Tukey post hoc test | U vs. NC:0.4728 U vs. - 27: <0.0001 NC vs. - 27: 0.0002 |
|  |  | 4.92 |  |  |  |  |
|  |  | 4.51 |  |  |  |  |
|  |  | 4.29 |  |  |  |  |
|  |  | 5.08 |  |  |  |  |
| Negative Control (NC) | 5 independent experiments (biological replicates), with monolayer cultures performed in triplicates (technical replicates) | 4.3 | 5.292 | 0.7531401 |  |  |
|  |  | 5.61 |  |  |  |  |
|  |  | 4.91 |  |  |  |  |
|  |  | 6.31 |  |  |  |  |
|  |  | 5.33 |  |  |  |  |
| miR-27a-3p Inhibitor (- 27) | 5 independent experiments (biological replicates), with monolayer cultures performed in triplicates (technical replicates) | 7.4 | 8.906 | 1.4586055 |  |  |
|  |  | 10.38 |  |  |  |  |
|  |  | 9.77 |  |  |  |  |
|  |  | 7.27 |  |  |  |  |
|  |  | 9.71 |  |  |  |  |
| **Fig 1E: Relative mRNA levels of junctions measured by PCR (2^-ΔΔCt^)** | | | | | | |
| Claudin-5 | | | | | | |
| Untreated (U) | 3 independent experiments (biological replicates), with PCR performed in duplicates (technical replicates) for each experiment | 0.745868 | 0.763209 | 0.030359 | One-way ANOVA followed by Tukey post hoc test | U vs. NC: 0.1905 U vs. - 27: 0.6897 NC vs. - 27: 0.515 |
|  |  | 0.745495 |  |  |  |  |
|  |  | 0.798263 |  |  |  |  |
| Negative Control (NC) |  | 1 | 1.000000 | 0.000000 |  |  |
|  |  | 1 |  |  |  |  |
|  |  | 1 |  |  |  |  |
| miR-27a-3p Inhibitor (- 27) |  | 1.047521 | 0.863063 | 0.248042 |  |  |
|  |  | 0.960586 |  |  |  |  |
|  |  | 0.581081 |  |  |  |  |
| Occludin | | | | | | |
| Untreated (U) | 3 independent experiments (biological replicates), with PCR performed in duplicates (technical replicates) for each experiment | 0.94678 | 0.983764 | 0.081171 | One-way ANOVA followed by Tukey post hoc test | U vs. NC: 0.9212 U vs. - 27: 0.9787 NC vs. - 27: 0.8321 |
|  |  | 1.07684 |  |  |  |  |
|  |  | 0.927671 |  |  |  |  |
| Negative Control (NC) |  | 1 | 1.000000 | 0.000000 |  |  |
|  |  | 1 |  |  |  |  |
|  |  | 1 |  |  |  |  |
| miR-27a-3p Inhibitor (- 27) |  | 0.936402 | 0.975492 | 0.035569 |  |  |
|  |  | 1.005952 |  |  |  |  |
|  |  | 0.984123 |  |  |  |  |
| VE-cadherin | | | | | | |
| Untreated (U) | 3 independent experiments (biological replicates), with PCR performed in duplicates (technical replicates) for each experiment | 0.967723 | 0.996654 | 0.025660 | One-way ANOVA followed by Tukey post hoc test | U vs. NC: 0.9928 U vs. - 27: 0.2711 NC vs. - 27: 0.2354 |
|  |  | 1.01666 |  |  |  |  |
|  |  | 1.005579 |  |  |  |  |
| Negative Control (NC) |  | 1 | 1.000000 | 0.000000 |  |  |
|  |  | 1 |  |  |  |  |
|  |  | 1 |  |  |  |  |
| miR-27a-3p Inhibitor (- 27) |  | 0.927675 | 0.946236 | 0.056376 |  |  |
|  |  | 0.90148 |  |  |  |  |
|  |  | 1.009552 |  |  |  |  |
| ZO-1 | | | | | | |
| Untreated (U) | 3 independent experiments (biological replicates), with PCR performed in duplicates (technical replicates) for each experiment | 1.055167 | 0.966147 | 0.086292 | One-way ANOVA followed by Tukey post hoc test | U vs. NC: 0.7267 U vs. - 27: 0.9403 NC vs. - 27 :0.8979 |
|  |  | 0.960402 |  |  |  |  |
|  |  | 0.882871 |  |  |  |  |
| Negative Control (NC) |  | 1 | 1.000000 | 0.000000 |  |  |
|  |  | 1 |  |  |  |  |
|  |  | 1 |  |  |  |  |
| miR-27a-3p Inhibitor (- 27) |  | 1.011399 | 0.980691 | 0.031359 |  |  |
|  |  | 0.981953 |  |  |  |  |
|  |  | 0.94872 |  |  |  |  |
| **Fig 1F: Normalized optical density of junctions measured by Western-Blot (Fold change relative to control )** | | | | | | |
| Claudin-5 | | | | | | |
| Untreated (U) | 3 independent experiments (biological replicates), with each preparation representing pooled protein lysates from monolayer cultures performed in triplicates (technical replicates) | 1.083846 | 1.118419 | 0.187787 | One-way ANOVA followed by Tukey post hoc test | U vs. NC: 0.4386 U vs. - 27: 0.0001 NC vs. - 27: 0.0003 |
|  |  | 1.321091 |  |  |  |  |
|  |  | 0.950321 |  |  |  |  |
| Negative Control (NC) |  | 1 | 1.000000 | 0.000000 |  |  |
|  |  | 1 |  |  |  |  |
|  |  | 1 |  |  |  |  |
| miR-27a-3p Inhibitor (- 27) |  | 0.173719 | 0.205333 | 0.035757 |  |  |
|  |  | 0.24414 |  |  |  |  |
|  |  | 0.198141 |  |  |  |  |
| Occludin | | | | | | |
| Untreated (U) | 3 independent experiments (biological replicates), with each preparation representing pooled protein lysates from monolayer cultures performed in triplicates (technical replicates) | 0.997345 | 0.997345 | #DIV/0! | One-way ANOVA followed by Tukey post hoc test | U vs. NC: 0.9962 U vs. - 27: <0.0001 NC vs. - 27: <0.0001 |
|  |  |  |  |  |  |  |
|  |  |  |  |  |  |  |
| Negative Control (NC) |  | 1 | 1.000000 | 0.000000 |  |  |
|  |  | 1 |  |  |  |  |
|  |  | 1 |  |  |  |  |
| miR-27a-3p Inhibitor (- 27) |  | 0.193781 | 0.236647 | 0.039283 |  |  |
|  |  | 0.270925 |  |  |  |  |
|  |  | 0.245236 |  |  |  |  |
| VE-cadherin | | | | | | |
| Untreated (U) | 3 independent experiments (biological replicates), with each preparation representing pooled protein lysates from monolayer cultures performed in triplicates (technical replicates) | 0.923363 | 0.881459 | 0.276714 | One-way ANOVA followed by Tukey post hoc test | U vs. NC: 0.8113 U vs. - 27: 0.3262 NC vs. - 27: 0.6338 |
|  |  | 1.134832 |  |  |  |  |
|  |  | 0.586183 |  |  |  |  |
| Negative Control (NC) |  | 1 | 1.000000 | 0.000000 |  |  |
|  |  | 1 |  |  |  |  |
|  |  | 1 |  |  |  |  |
| miR-27a-3p Inhibitor (- 27) |  | 1.504674 | 1.178857 | 0.290232 |  |  |
|  |  | 1.083895 |  |  |  |  |
|  |  | 0.948001 |  |  |  |  |
| ZO-1 | | | | | | |
| Untreated (U) | 3 independent experiments (biological replicates), with each preparation representing pooled protein lysates from monolayer cultures performed in triplicates (technical replicates) | 0.798122 | 1.012985 | 0.187191 | One-way ANOVA followed by Tukey post hoc test | U vs. NC: 0.9938 U vs. - 27: 0.9441 NC vs. - 27: 0.9042 |
|  |  | 1.100023 |  |  |  |  |
|  |  | 1.140809 |  |  |  |  |
| Negative Control (NC) |  | 1 | 1.000000 | 0.000000 |  |  |
|  |  | 1 |  |  |  |  |
|  |  | 1 |  |  |  |  |
| miR-27a-3p Inhibitor (- 27) |  | 1.036505 | 1.052589 | 0.178642 |  |  |
|  |  | 0.882532 |  |  |  |  |
|  |  | 1.238729 |  |  |  |  |

**Fig 2**

| **Figure Number** | **Number of Experiments** | **Individual values** | **Mean** | **S.D.** | **Statistical method used** | ***p* value** |
| --- | --- | --- | --- | --- | --- | --- |
| **Fig 2A: Relative miR-27a-3p levels measured by PCR (2^-ΔCt^)** | | | | | | |
| Untreated (U) | 3 independent experiments (biological replicates), with PCR performed in duplicates (technical replicates) for each experiment | 1.0326 | 0.908433 | 0.119817 | One-way ANOVA followed by Tukey post hoc test | U vs. C: 0.7756 U vs. + 27: 0.0045 C vs. + 27: 0.009 |
|  |  | 0.7935 |  |  |  |  |
|  |  | 0.8992 |  |  |  |  |
| Control (C) |  | 2.331022 | 2.470343 | 0.194473 |  |  |
|  |  | 2.692522 |  |  |  |  |
|  |  | 2.387486 |  |  |  |  |
| miR-27a-3p Mimic (+ 27) |  | 18.23881 | 12.760631 | 4.768611 |  |  |
|  |  | 10.50302 |  |  |  |  |
|  |  | 9.540063 |  |  |  |  |
| **Fig 2B: TEER (Ω.cm^2^)** | | | | | | |
| Untreated (U) | 6 independent experiments (biological replicates), with monolayer cultures performed in triplicates (technical replicates) | 72 | 66.833333 | 12.05681 | One-way ANOVA followed by Tukey post hoc test | U vs. C: 0.0647 U vs. + 27: 0.0038 C vs. + 27: <0.0001 |
|  |  | 79 |  |  |  |  |
|  |  | 61 |  |  |  |  |
|  |  | 80 |  |  |  |  |
|  |  | 59 |  |  |  |  |
|  |  | 50 |  |  |  |  |
| Control (C) |  | 53 | 52.000000 | 12.312595 |  |  |
|  |  | 61 |  |  |  |  |
|  |  | 39 |  |  |  |  |
|  |  | 35 |  |  |  |  |
|  |  | 65 |  |  |  |  |
|  |  | 59 |  |  |  |  |
| miR-27a-3p Mimic (+ 27) |  | 85 | 90.333333 | 5.5015149 |  |  |
|  |  | 92 |  |  |  |  |
|  |  | 90 |  |  |  |  |
|  |  | 95 |  |  |  |  |
|  |  | 97 |  |  |  |  |
|  |  | 83 |  |  |  |  |
| **Fig 2C: Pe_4kDa_ A/B (10^-6^ cm/s)** | | | | | | |
| Untreated (U) | 5 independent experiments (biological replicates), with monolayer cultures performed in triplicates (technical replicates) | 6.46 | 10.864 | 3.4014747 | One-way ANOVA followed by Tukey post hoc test | U vs. C: 0.6008 U vs. + 27: 0.0633 C vs. + 27: 0.011 |
|  |  | 12.22 |  |  |  |  |
|  |  | 8.25 |  |  |  |  |
|  |  | 12.65 |  |  |  |  |
|  |  | 14.74 |  |  |  |  |
| Control (C) | 5 independent experiments (biological replicates), with monolayer cultures performed in triplicates (technical replicates) | 7.16 | 13.164 | 5.2515264 |  |  |
|  |  | 12.7 |  |  |  |  |
|  |  | 9 |  |  |  |  |
|  |  | 18.79 |  |  |  |  |
|  |  | 18.17 |  |  |  |  |
| miR-27a-3p Mimic (+ 27) | 5 independent experiments (biological replicates), with monolayer cultures performed in triplicates (technical replicates) | 3.94 | 4.938 | 1.3801341 |  |  |
|  |  | 6.01 |  |  |  |  |
|  |  | 3.11 |  |  |  |  |
|  |  | 6.36 |  |  |  |  |
|  |  | 5.27 |  |  |  |  |
| **Fig 2D: Pe_70kDa_ A/B (10^-6^ cm/s)** | | | | | | |
| Untreated (U) | 5 independent experiments (biological replicates), with monolayer cultures performed in triplicates (technical replicates) | 3 | 4.444 | 0.9386853 | One-way ANOVA followed by Tukey post hoc test | U vs. C: 0.8104 U vs. + 27: 0.0042 C vs. + 27: 0.0014 |
|  |  | 4.95 |  |  |  |  |
|  |  | 4.21 |  |  |  |  |
|  |  | 4.56 |  |  |  |  |
|  |  | 5.5 |  |  |  |  |
| Control (C) | 5 independent experiments (biological replicates), with monolayer cultures performed in triplicates (technical replicates) | 3.76 | 4.798 | 1.0469814 |  |  |
|  |  | 4.35 |  |  |  |  |
|  |  | 4.81 |  |  |  |  |
|  |  | 4.53 |  |  |  |  |
|  |  | 6.54 |  |  |  |  |
| miR-27a-3p Mimic (+ 27) | 5 independent experiments (biological replicates), with monolayer cultures performed in triplicates (technical replicates) | 1.6 | 2.14 | 0.6648308 |  |  |
|  |  | 2.72 |  |  |  |  |
|  |  | 2.48 |  |  |  |  |
|  |  | 1.26 |  |  |  |  |
|  |  | 2.64 |  |  |  |  |
| **Fig 2E: Relative mRNA levels of junctions measured by PCR (2^-ΔΔCt^)** | | | | | | |
| Claudin-5 | | | | | | |
| Untreated (U) | 3 independent experiments (biological replicates), with PCR performed in duplicates (technical replicates) for each experiment | 0.70255 | 0.714241 | 0.108603 | One-way ANOVA followed by Tukey post hoc test | U vs. C: 0.1308 U vs. + 27: 0.1677 C vs. + 27: 0.9793 |
|  |  | 0.611956 |  |  |  |  |
|  |  | 0.828216 |  |  |  |  |
| Control (C) |  | 1 | 1.000000 | 0.000000 |  |  |
|  |  | 1 |  |  |  |  |
|  |  | 1 |  |  |  |  |
| miR-27a-3p Mimic (+ 27) |  | 0.804903 | 0.975846 | 0.239414 |  |  |
|  |  | 0.873162 |  |  |  |  |
|  |  | 1.249474 |  |  |  |  |
| Occludin | | | | | | |
| Untreated (U) | 3 independent experiments (biological replicates), with PCR performed in duplicates (technical replicates) for each experiment | 0.877101 | 0.922521 | 0.039385 | One-way ANOVA followed by Tukey post hoc test | U vs. C: 0.1012 U vs. + 27: 0.9998 C vs. + 27:0.0989 |
|  |  | 0.947216 |  |  |  |  |
|  |  | 0.943247 |  |  |  |  |
| Control (C) |  | 1 | 1.000000 | 0.000000 |  |  |
|  |  | 1 |  |  |  |  |
|  |  | 1 |  |  |  |  |
| miR-27a-3p Mimic (+ 27) |  | 0.890424 | 0.921961 | 0.052413 |  |  |
|  |  | 0.892995 |  |  |  |  |
|  |  | 0.982464 |  |  |  |  |
| VE-cadherin | | | | | | |
| Untreated (U) | 3 independent experiments (biological replicates), with PCR performed in duplicates (technical replicates) for each experiment | 1.039444 | 0.948874 | 0.087296 | One-way ANOVA followed by Tukey post hoc test | U vs. C: 0.8815 U vs. + 27: 0.7473 C vs. + 27: 0.9631 |
|  |  | 0.94191 |  |  |  |  |
|  |  | 0.865269 |  |  |  |  |
| Control (C) |  | 1 | 1.000000 | 0.000000 |  |  |
|  |  | 1 |  |  |  |  |
|  |  | 1 |  |  |  |  |
| miR-27a-3p Mimic (+ 27) |  | 0.998114 | 1.027704 | 0.206649 |  |  |
|  |  | 1.247553 |  |  |  |  |
|  |  | 0.837445 |  |  |  |  |
| ZO-1 | | | | | | |
| Untreated (U) | 3 independent experiments (biological replicates), with PCR performed in duplicates (technical replicates) for each experiment | 0.932588 | 0.963661 | 0.034868 | One-way ANOVA followed by Tukey post hoc test | U vs. C: 0.2906 U vs. + 27: >0.9999 C vs. + 27: 0.2883 |
|  |  | 0.957025 |  |  |  |  |
|  |  | 1.00137 |  |  |  |  |
| Control (C) |  | 1 | 1.000000 | 0.000000 |  |  |
|  |  | 1 |  |  |  |  |
|  |  | 1 |  |  |  |  |
| miR-27a-3p Mimic (+ 27) |  | 0.932233 | 0.963514 | 0.030267 |  |  |
|  |  | 0.992654 |  |  |  |  |
|  |  | 0.965654 |  |  |  |  |
| **Fig 2F: Normalized optical density of junctions measured by Western-Blot (Fold change relative to control )** | | | | | | |
| Claudin-5 | | | | | | |
| Untreated (U) | 3 independent experiments (biological replicates), with each preparation representing pooled protein lysates from monolayer cultures performed in triplicates (technical replicates) | 1.048332 | 1.000345 | 0.091151 | One-way ANOVA followed by Tukey post hoc test | U vs. C: >0.9999 U vs. + 27: 0.0118 C vs. + 27: 0.0117 |
|  |  | 0.895225 |  |  |  |  |
|  |  | 1.057478 |  |  |  |  |
| Control (C) |  | 1 | 1.000000 | 0.000000 |  |  |
|  |  | 1 |  |  |  |  |
|  |  | 1 |  |  |  |  |
| miR-27a-3p Mimic (+ 27) |  | 1.565027 | 1.599196 | 0.279341 |  |  |
|  |  | 1.338512 |  |  |  |  |
|  |  | 1.89405 |  |  |  |  |
| Occludin | | | | | | |
| Untreated (U) | 3 independent experiments (biological replicates), with each preparation representing pooled protein lysates from monolayer cultures performed in triplicates (technical replicates) | 0.912134 | 0.846488 | 0.066992 | One-way ANOVA followed by Tukey post hoc test | U vs. C: 0.8611 U vs. + 27: 0.0032 C vs. + 27:0.0052 |
|  |  | 0.849104 |  |  |  |  |
|  |  | 0.778226 |  |  |  |  |
| Control (C) |  | 1 | 1.000000 | 0.000000 |  |  |
|  |  | 1 |  |  |  |  |
|  |  | 1 |  |  |  |  |
| miR-27a-3p Mimic (+ 27) |  | 2.980798 | 2.488763 | 0.613535 |  |  |
|  |  | 2.684165 |  |  |  |  |
|  |  | 1.801325 |  |  |  |  |
| VE-cadherin | | | | | | |
| Untreated (U) | 3 independent experiments (biological replicates), with each preparation representing pooled protein lysates from monolayer cultures performed in triplicates (technical replicates) | 0.985418 | 0.969991 | 0.278302 | One-way ANOVA followed by Tukey post hoc test | U vs. C: 0.9904 U vs. + 27: 0.9961 C vs. + 27: 0.9987 |
|  |  | 1.240259 |  |  |  |  |
|  |  | 0.684296 |  |  |  |  |
| Control (C) |  | 1 | 1.000000 | 0.000000 |  |  |
|  |  | 1 |  |  |  |  |
|  |  | 1 |  |  |  |  |
| miR-27a-3p Mimic (+ 27) |  | 1.205981 | 0.988941 | 0.390921 |  |  |
|  |  | 1.223189 |  |  |  |  |
|  |  | 0.537654 |  |  |  |  |
| ZO-1 | | | | | | |
| Untreated (U) | 3 independent experiments (biological replicates), with each preparation representing pooled protein lysates from monolayer cultures performed in triplicates (technical replicates) | 1.258803 | 1.190506 | 0.159155 | One-way ANOVA followed by Tukey post hoc test | U vs. C: 0.1955 U vs. + 27: 0.5911 C vs. + 27: 0.6217 |
|  |  | 1.304113 |  |  |  |  |
|  |  | 1.008601 |  |  |  |  |
| Control (C) |  | 1 | 1.000000 | 0.000000 |  |  |
|  |  | 1 |  |  |  |  |
|  |  | 1 |  |  |  |  |
| miR-27a-3p Mimic (+ 27) |  | 1.129867 | 1.092632 | 0.126179 |  |  |
|  |  | 1.196004 |  |  |  |  |
|  |  | 0.952026 |  |  |  |  |

**Fig 3**

| **Figure Number** | **Number of Experiments** | **Individual values** | **Mean** | **S.D.** | **Statistical method used** | ***p* value** |
| --- | --- | --- | --- | --- | --- | --- |
| **Fig 3B: Fold change of luciferase activity (Firefly/Renilla)** | | | | | | |
| pmiR-GLO- GSK3B-3'UTR-WT: Control (C) | 3 independent experiments (biological replicates), with tests performed in triplicates (technical replicates) for each experiment | 1 | 1.000000 | 0.000000 | Paired t test | C vs. + 27: 0.0222 |
|  |  | 1 |  |  |  |  |
|  |  | 1 |  |  |  |  |
| pmiR-GLO- GSK3B-3'UTR-WT: miR-27a-3p Mimic (+ 27) |  | 0.446484 | 0.453315 | 0.143349 |  |  |
|  |  | 0.599958 |  |  |  |  |
|  |  | 0.313504 |  |  |  |  |
| pmiR-GLO- GSK3B-3'UTR-MUT: Control (C) | 3 independent experiments (biological replicates), with tests performed in triplicates (technical replicates) for each experiment | 1 | 1.000000 | 0.000000 | Paired t test | C vs. + 27: 0.1119 |
|  |  | 1 |  |  |  |  |
|  |  | 1 |  |  |  |  |
| pmiR-GLO- GSK3B-3'UTR-MUT: miR-27a-3p Mimic (+ 27) |  | 0.927247 | 0.904628 | 0.060450 |  |  |
|  |  | 0.950506 |  |  |  |  |
|  |  | 0.83613 |  |  |  |  |
| **Fig 3C: Relative GSK3B mRNA levels measured by PCR (2^-ΔCt^)** | | | | | | |
| Untreated (U) | 3 independent experiments (biological replicates), with PCR performed in duplicates (technical replicates) for each experiment | 0.041365 | 0.034117 | 0.007304 | One-way ANOVA followed by Tukey post hoc test | U vs. C: 0.0565 U vs. + 27: 0.0085 C vs. + 27: 0.0007 |
|  |  | 0.034228 |  |  |  |  |
|  |  | 0.026759 |  |  |  |  |
| Control (C) |  | 0.044886 | 0.052730 | 0.011000 |  |  |
|  |  | 0.065304 |  |  |  |  |
|  |  | 0.048 |  |  |  |  |
| miR-27a-3p Mimic (+ 27) |  | 0.00555 | 0.005087 | 0.001560 |  |  |
|  |  | 0.003348 |  |  |  |  |
|  |  | 0.006363 |  |  |  |  |
| **Fig 3D: Relative B-catenin mRNA levels measured by PCR (2^-ΔCt^)** | | | | | | |
| Untreated (U) | 3 independent experiments (biological replicates), with PCR performed in duplicates (technical replicates) for each experiment | 0.00133 | 0.001110 | 0.000208 | One-way ANOVA followed by Tukey post hoc test | U vs. C: 0.9889 U vs. + 27: 0.0001 C vs. + 27: 0.0001 |
|  |  | 0.000916 |  |  |  |  |
|  |  | 0.001085 |  |  |  |  |
| Control (C) |  | 0.00055 | 0.000954 | 0.000362 |  |  |
|  |  | 0.001249 |  |  |  |  |
|  |  | 0.001064 |  |  |  |  |
| miR-27a-3p Mimic (+ 27) |  | 0.011817 | 0.012478 | 0.002286 |  |  |
|  |  | 0.010596 |  |  |  |  |
|  |  | 0.015022 |  |  |  |  |
| **Fig 3E: Normalized optical density of GSK3ß measured by Western-Blot (Fold change relative to control )** | | | | | | |
| Untreated (U) | 6 independent experiments (biological replicates), with each preparation representing pooled protein lysates from monolayer cultures performed in triplicates (technical replicates) | 1.475692 | 1.050292 | 0.2626122 | One-way ANOVA followed by Tukey post hoc test | U vs. C: 0.8556 U vs. + 27: 0.0026 C vs. + 27: 0.0077 |
|  |  | 0.833897 |  |  |  |  |
|  |  | 1.265418 |  |  |  |  |
|  |  | 0.825612 |  |  |  |  |
|  |  | 0.936729 |  |  |  |  |
|  |  | 0.964403 |  |  |  |  |
| Control (C) |  | 1 | 1.000000 | 0 |  |  |
|  |  | 1 |  |  |  |  |
|  |  | 1 |  |  |  |  |
|  |  | 1 |  |  |  |  |
|  |  | 1 |  |  |  |  |
|  |  | 1 |  |  |  |  |
| miR-27a-3p Mimic (+ 27) |  | 0.602463 | 0.665900 | 0.1031988 |  |  |
|  |  | 0.737628 |  |  |  |  |
|  |  | 0.60097 |  |  |  |  |
|  |  | 0.544935 |  |  |  |  |
|  |  | 0.685923 |  |  |  |  |
|  |  | 0.82348 |  |  |  |  |
| **Fig 3F: Normalized optical density of nuclear ß-catenin measured by Western-Blot (Fold change relative to control )** | | | | | | |
| Untreated (U) | 3 independent experiments (biological replicates), with each preparation representing pooled protein lysates from monolayer cultures performed in triplicates (technical replicates) | 1.481904 | 1.108630 | 0.339775 | One-way ANOVA followed by Tukey post hoc test | U vs. C: 0.9082 U vs. + 27: 0.0793 C vs. + 27: 0.0469 |
|  |  | 1.026621 |  |  |  |  |
|  |  | 0.817365 |  |  |  |  |
| Control (C) |  | 1 | 1.000000 | 0.000000 |  |  |
|  |  | 1 |  |  |  |  |
|  |  | 1 |  |  |  |  |
| miR-27a-3p Mimic (+ 27) |  | 1.548206 | 1.804647 | 0.428636 |  |  |
|  |  | 2.299484 |  |  |  |  |
|  |  | 1.566251 |  |  |  |  |

**Fig 4**

| **Figure Number** | **Number of Experiments** | **Individual values** | **Mean** | **S.D.** | **Statistical method used** | ***p* value** |
| --- | --- | --- | --- | --- | --- | --- |
| **Fig 4A: Normalized optical density of GSK3β, β-catenin, claudin-5 and occludin measured by Western-Blot (Fold change relative to control )** | | | | | | |
| GSK3β | | | | | | |
| Negative Control (NC) | 4 independent experiments (biological replicates), with each preparation representing pooled protein lysates from monolayer cultures performed in triplicates (technical replicates) | 1 | 1.000000 | 0.000000 | One-way ANOVA followed by Tukey post hoc test | NC vs. - 27: 0.0004 NC vs. - GSK3β: 0.0022 NC vs. - 27 - GSK3β: 0.0042 - 27 vs. - GSK3β: <0.0001 - 27 vs. - 27 - GSK3β: <0.0001 - GSK3β vs. - 27 - GSK3β: 0.9782 |
|  |  | 1 |  |  |  |  |
|  |  | 1 |  |  |  |  |
|  |  | 1 |  |  |  |  |
| miR-27a-3p Inhibitor (- 27) |  | 1.363442 | 1.587513 | 0.277193 |  |  |
|  |  | 1.817501 |  |  |  |  |
|  |  | 1.836931 |  |  |  |  |
|  |  | 1.332176 |  |  |  |  |
| GSK3β Inhibitor (- GSK3β) |  | 0.527268 | 0.513988 | 0.057552 |  |  |
|  |  | 0.472244 |  |  |  |  |
|  |  | 0.589915 |  |  |  |  |
|  |  | 0.466525 |  |  |  |  |
| miR-27a-3p Inhibitor + GSK3β Inhibitor (-27a - GSK3β) |  | 0.512701 | 0.554048 | 0.048006 |  |  |
|  |  | 0.62207 |  |  |  |  |
|  |  | 0.551234 |  |  |  |  |
|  |  | 0.530188 |  |  |  |  |
| nuclear β-catenin | | | | | | |
| Negative Control (NC) | 4 independent experiments (biological replicates), with each preparation representing pooled protein lysates from monolayer cultures performed in triplicates (technical replicates) | 1 | 1.000000 | 0.000000 | One-way ANOVA followed by Tukey post hoc test | NC vs. - 27: 0.0107 NC vs. - GSK3β: 0.0002 NC vs. - 27 - GSK3β: <0.0001 - 27 vs. - GSK3β: <0.0001 - 27 vs. - 27 - GSK3β: <0.0001 - GSK3β vs. - 27 - GSK3β: 0.9261 |
|  |  | 1 |  |  |  |  |
|  |  | 1 |  |  |  |  |
|  |  | 1 |  |  |  |  |
| miR-27a-3p Inhibitor (- 27) |  | 0.526888 | 0.569766 | 0.097309 |  |  |
|  |  | 0.644107 |  |  |  |  |
|  |  | 0.452676 |  |  |  |  |
|  |  | 0.655392 |  |  |  |  |
| GSK3β Inhibitor (- GSK3β) |  | 1.521102 | 1.698031 | 0.241512 |  |  |
|  |  | 1.855136 |  |  |  |  |
|  |  | 1.464219 |  |  |  |  |
|  |  | 1.951665 |  |  |  |  |
| miR-27a-3p Inhibitor + GSK3β Inhibitor (-27a - GSK3β) |  | 1.978794 | 1.766449 | 0.178729 |  |  |
|  |  | 1.616215 |  |  |  |  |
|  |  | 1.849942 |  |  |  |  |
|  |  | 1.620846 |  |  |  |  |
| Claudin-5 | | | | | | |
| Negative Control (NC) | 4 independent experiments (biological replicates), with each preparation representing pooled protein lysates from monolayer cultures performed in triplicates (technical replicates) | 1 | 1.000000 | 0.000000 | One-way ANOVA followed by Tukey post hoc test | NC vs. - 27: 0.0138 NC vs. - GSK3β: 0.0583 NC vs. - 27 - GSK3β: 0.0037 - 27 vs. - GSK3β: 0.0001 - 27 vs. - 27 - GSK3β: <0.0001 - GSK3β vs. - 27 - GSK3β: 0.4173 |
|  |  | 1 |  |  |  |  |
|  |  | 1 |  |  |  |  |
|  |  | 1 |  |  |  |  |
| miR-27a-3p Inhibitor (- 27) |  | 0.39249 | 0.322659 | 0.057228 |  |  |
|  |  | 0.346414 |  |  |  |  |
|  |  | 0.277488 |  |  |  |  |
|  |  | 0.274244 |  |  |  |  |
| GSK3β Inhibitor (- GSK3β) |  | 1.346757 | 1.526539 | 0.133063 |  |  |
|  |  | 1.631163 |  |  |  |  |
|  |  | 1.623386 |  |  |  |  |
|  |  | 1.50485 |  |  |  |  |
| miR-27a-3p Inhibitor + GSK3β Inhibitor (-27a - GSK3β) |  | 1.458939 | 1.818155 | 0.496560 |  |  |
|  |  | 2.550116 |  |  |  |  |
|  |  | 1.683944 |  |  |  |  |
|  |  | 1.579621 |  |  |  |  |
| Occludin | | | | | | |
| Negative Control (NC) | 3 independent experiments (biological replicates), with each preparation representing pooled protein lysates from monolayer cultures performed in triplicates (technical replicates) | 1 | 1.000000 | 0.000000 | One-way ANOVA followed by Tukey post hoc test | NC vs. - 27: 0.1549 NC vs. - GSK3β: 0.0001 NC vs. - 27 - GSK3β: <0.0001 - 27 vs. - GSK3β: <0.0001 - 27 vs. - 27 - GSK3β: <0.0001 - GSK3β vs. - 27 - GSK3β: 0.7407 |
|  |  | 1 |  |  |  |  |
|  |  | 1 |  |  |  |  |
| miR-27a-3p Inhibitor (- 27) |  | 0.193781 | 0.236647 | 0.039283 |  |  |
|  |  | 0.270925 |  |  |  |  |
|  |  | 0.245236 |  |  |  |  |
| GSK3β Inhibitor (- GSK3β) |  | 4.16066 | 3.776932 | 0.429231 |  |  |
|  |  | 3.856736 |  |  |  |  |
|  |  | 3.3134 |  |  |  |  |
| miR-27a-3p Inhibitor + GSK3β Inhibitor (-27a - GSK3β) |  | 4.37359 | 4.103167 | 0.650000 |  |  |
|  |  | 4.5743 |  |  |  |  |
|  |  | 3.361611 |  |  |  |  |
| **Fig 4B: TEER (Ω.cm^2^)** | | | | | | |
| Untreated (U) | 6 independent experiments (biological replicates), with monolayer cultures performed in triplicates (technical replicates) | 72 | 66.833333 | 12.05681 | One-way ANOVA followed by Bonferroni post hoc test | U vs. NC: 0.0034 U vs. - 27: <0.0001 U vs. - GSK3β: 0.0089 U vs. - 27 - GSK3β: 0.0011 NC vs. - 27: 0.0001 NC vs. - GSK3β: >0.9999 NC vs. - 27 - GSK3β: >0.9999 - 27 vs. - GSK3β: <0.0001 - 27 vs. - 27 - GSK3β: 0.0004 - GSK3β vs. - 27 - GSK3β: >0.9999 |
|  |  | 79 |  |  |  |  |
|  |  | 61 |  |  |  |  |
|  |  | 80 |  |  |  |  |
|  |  | 59 |  |  |  |  |
|  |  | 50 |  |  |  |  |
| Negative Control (NC) |  | 57 | 49.000000 | 10.807405 |  |  |
|  |  | 66 |  |  |  |  |
|  |  | 42 |  |  |  |  |
|  |  | 39 |  |  |  |  |
|  |  | 40 |  |  |  |  |
|  |  | 50 |  |  |  |  |
| miR-27a-3p Inhibitor (- 27) |  | 26 | 19.833333 | 5.5647701 |  |  |
|  |  | 19 |  |  |  |  |
|  |  | 15 |  |  |  |  |
|  |  | 25 |  |  |  |  |
|  |  | 12 |  |  |  |  |
|  |  | 22 |  |  |  |  |
| GSK3β Inhibitor (- GSK3β) |  | 26 | 19.833333 | 5.5647701 |  |  |
|  |  | 19 |  |  |  |  |
|  |  | 15 |  |  |  |  |
|  |  | 25 |  |  |  |  |
|  |  | 12 |  |  |  |  |
|  |  | 22 |  |  |  |  |
| miR-27a-3p Inhibitor + GSK3β Inhibitor (-27a - GSK3β) |  | 26 | 19.833333 | 5.5647701 |  |  |
|  |  | 19 |  |  |  |  |
|  |  | 15 |  |  |  |  |
|  |  | 25 |  |  |  |  |
|  |  | 12 |  |  |  |  |
|  |  | 22 |  |  |  |  |
| **Fig 4C: Pe_4kDa_ A/B (10^-6^ cm/s)** | | | | | | |
| Negative Control (NC) | 5 independent experiments (biological replicates), with monolayer cultures performed in triplicates (technical replicates) | 4.3 | 5.292000 | 0.7531401 | One-way ANOVA followed by Tukey post hoc test | NC vs. - GSK3β: 0.4652 NC vs. - 27 - GSK3β: 0.9927 - GSK3β vs. - 27 - GSK3β: 0.5301 |
|  |  | 5.61 |  |  |  |  |
|  |  | 4.91 |  |  |  |  |
|  |  | 6.31 |  |  |  |  |
|  |  | 5.33 |  |  |  |  |
| GSK3β Inhibitor (- GSK3β) |  | 3 | 3.534000 | 0.5207975 |  |  |
|  |  | 3.76 |  |  |  |  |
|  |  | 3.91 |  |  |  |  |
|  |  | 2.95 |  |  |  |  |
|  |  | 4.05 |  |  |  |  |
| miR-27a-3p Inhibitor + GSK3β Inhibitor |  | 2.8 | 3.554000 | 0.7279286 |  |  |
|  |  | 4.71 |  |  |  |  |
|  |  | 3.52 |  |  |  |  |
|  |  | 3.1 |  |  |  |  |
|  |  | 3.64 |  |  |  |  |
| **Fig 4D: Pe_70kDa_ A/B (10^-6^ cm/s)** | | | | | | |
| Negative Control (NC) | 5 independent experiments (biological replicates), with monolayer cultures performed in triplicates (technical replicates) | 6.81 | 8.682000 | 2.1480968 | One-way ANOVA followed by Tukey post hoc test | NC vs. - GSK3β: 0.0038 NC vs. - 27 - GSK3β: 0.0041 - GSK3β vs. - 27 - GSK3β: 0.9988 |
|  |  | 11.35 |  |  |  |  |
|  |  | 8.67 |  |  |  |  |
|  |  | 6.35 |  |  |  |  |
|  |  | 10.23 |  |  |  |  |
| GSK3β Inhibitor (- GSK3β) |  | 4.29 | 6.570000 | 1.9160767 |  |  |
|  |  | 8.62 |  |  |  |  |
|  |  | 6.83 |  |  |  |  |
|  |  | 4.94 |  |  |  |  |
|  |  | 8.17 |  |  |  |  |
| miR-27a-3p Inhibitor + GSK3β Inhibitor |  | 4.12 | 8.482000 | 3.7732506 |  |  |
|  |  | 11.74 |  |  |  |  |
|  |  | 7.22 |  |  |  |  |
|  |  | 6.28 |  |  |  |  |
|  |  | 13.05 |  |  |  |  |

**Fig S1**

| **Figure Number** | **Number of Experiments** | **Individual values** | **Mean** | **S.D.** | **Statistical method used** | ***p* value** |
| --- | --- | --- | --- | --- | --- | --- |
| **Fig S1A: Normalized optical density of β-catenin in the membrane fractions measured by Western-Blot (Fold change relative to control )** | | | | | | |
| Negative Control (NC) | 3 independent experiments (biological replicates), with each preparation representing pooled protein lysates from monolayer cultures performed in triplicates (technical replicates) | 1 | 1.000000 | 0.000000 | One-way ANOVA followed by Tukey post hoc test | NC vs. - 27: 0.3158 NC vs. - GSK3β: 0.0463 NC vs. - 27 - GSK3β: 0.1137 - 27 vs. - GSK3β: 0.004 - 27 vs. - 27 - GSK3β: 0.0089 - GSK3β vs. - 27 - GSK3β: 0.9169 |
|  |  | 1 |  |  |  |  |
|  |  | 1 |  |  |  |  |
| miR-27a-3p Inhibitor (- 27) |  | 0.155026 | 0.122285 | 0.041002 |  |  |
|  |  | 0.135531 |  |  |  |  |
|  |  | 0.076297 |  |  |  |  |
| GSK3β Inhibitor (- GSK3β) |  | 3.532863 | 2.537987 | 0.951966 |  |  |
|  |  | 2.4454 |  |  |  |  |
|  |  | 1.635697 |  |  |  |  |
| miR-27a-3p Inhibitor + GSK3β Inhibitor (-27a - GSK3β) |  | 2.935373 | 2.236994 | 0.655351 |  |  |
|  |  | 2.140163 |  |  |  |  |
|  |  | 1.635445 |  |  |  |  |
| **Fig S1B: Normalized optical density of β-catenin in the membrane fractions measured by Western-Blot (Fold change relative to control )** | | | | | | |
| Untreated (U) | 3 independent experiments (biological replicates), with each preparation representing pooled protein lysates from monolayer cultures performed in triplicates (technical replicates) |  | 0.968432 | 0.029566 | One-way ANOVA followed by Tukey post hoc test | U vs. C: 0.9818 U vs. + 27: 0.0023 C vs. + 27: 0.0015 |
|  |  | 0.947526 |  |  |  |  |
|  |  | 0.989338 |  |  |  |  |
| Control (C) |  | 1 | 1.000000 | 0.000000 |  |  |
|  |  | 1 |  |  |  |  |
|  |  | 1 |  |  |  |  |
| miR-27a-3p Mimic (+ 27) |  | 2.184772 | 2.160056 | 0.298290 |  |  |
|  |  | 2.445219 |  |  |  |  |
|  |  | 1.850176 |  |  |  |  |
