# Supplementary material for: miR-27a-3p regulates expression of intercellular junctions at the brain endothelium and controls the endothelial barrier permeability
Source: PLoS One. 2022 Jan 13;17(1):e0262152. doi: 10.1371/journal.pone.0262152 (PMC8758013; doi:10.1371/journal.pone.0262152)
Supplement: S1 Raw images — (DOCX) [file pone.0262152.s003.docx]

**miR-27a-3p regulates expression of intercellular junctions at the brain endothelium and controls the endothelial barrier permeability**

**Short Title: Regulation of inter-endothelial junctions by miR-27a-3p**

**Rania Harati^1,2*^, Saba Hammad^1,2^, Abdelaziz Tlili^3^, Mona Mahfood^3^, Aloïse Mabondzo^4^ & Rifat Hamoudi^2,5,6^**

^1^Department of Pharmacy Practice and Pharmacotherapeutics, College of Pharmacy, University of Sharjah, 27272, Sharjah, United Arab Emirates

^2^Sharjah Institute for Medical Research, University of Sharjah, 27272, Sharjah, United Arab Emirates

^3^Department of Applied Biology, College of Sciences, University of Sharjah, 27272, Sharjah, United Arab Emirates

^4^Paris-Saclay University, Department of Medicines and Healthcare Technologies, The French Alternative Energies and Atomic Energy Commission, 91191, Gif-sur-Yvette, France

^5^Clinical Sciences Department, College of Medicine, University of Sharjah, 27272, Sharjah, United Arab Emirates

^6^Division of Surgery and Interventional Science, University College London, W1W 7EJ London, United Kingdom

Corresponding author: Rania Harati; [rharati@sharjah.ac.ae](mailto:rharati@sharjah.ac.ae)

**Western Blot Images**

**
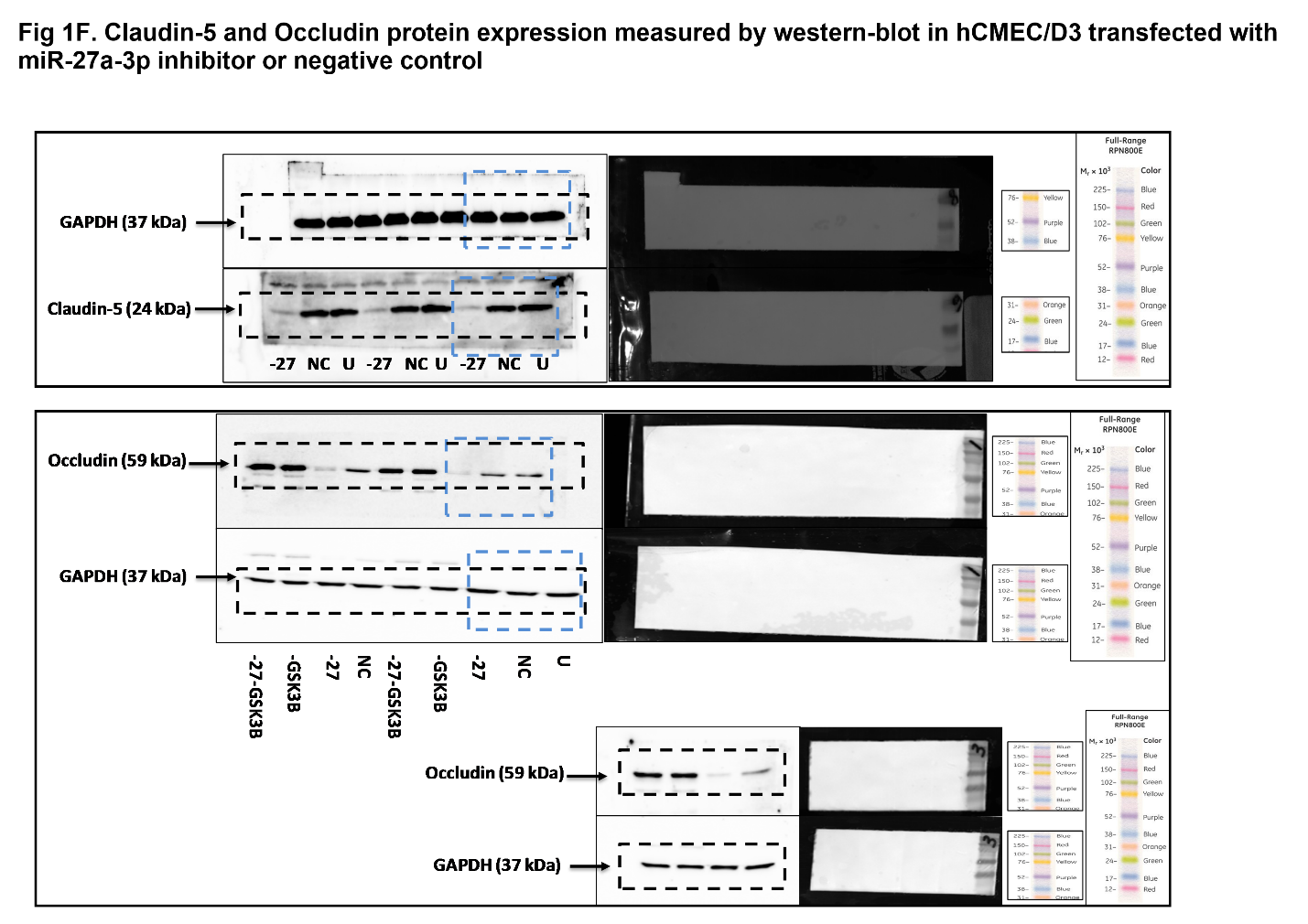

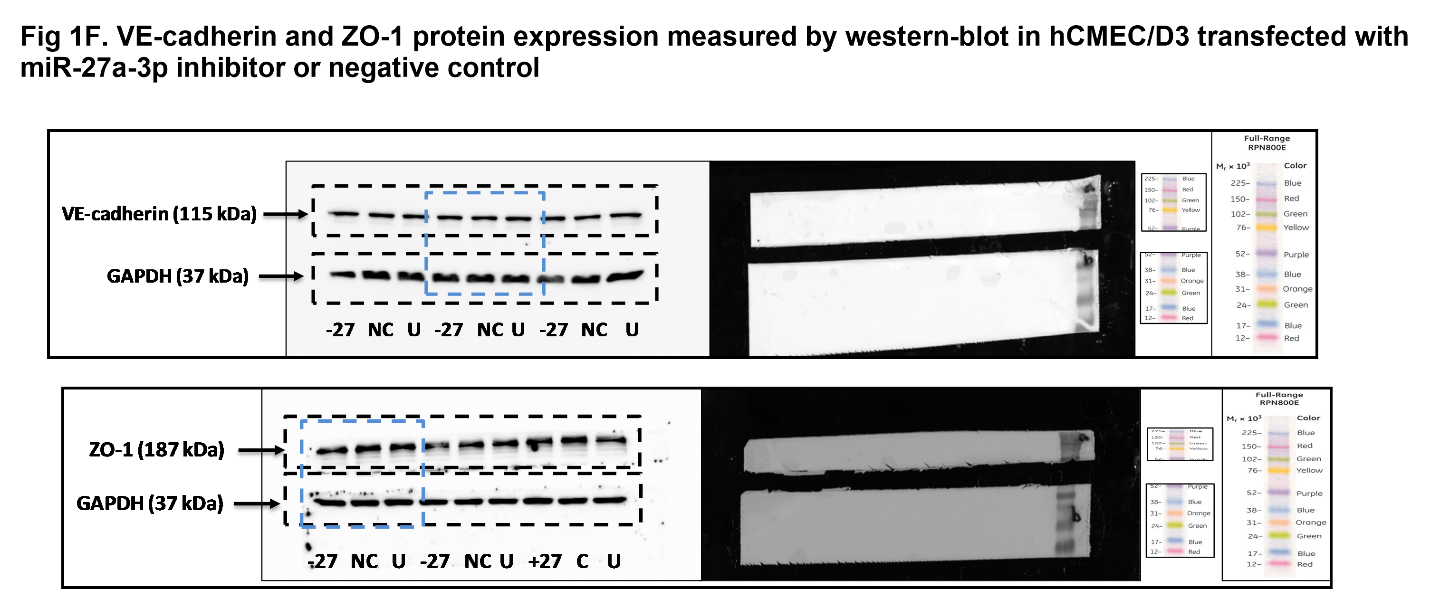

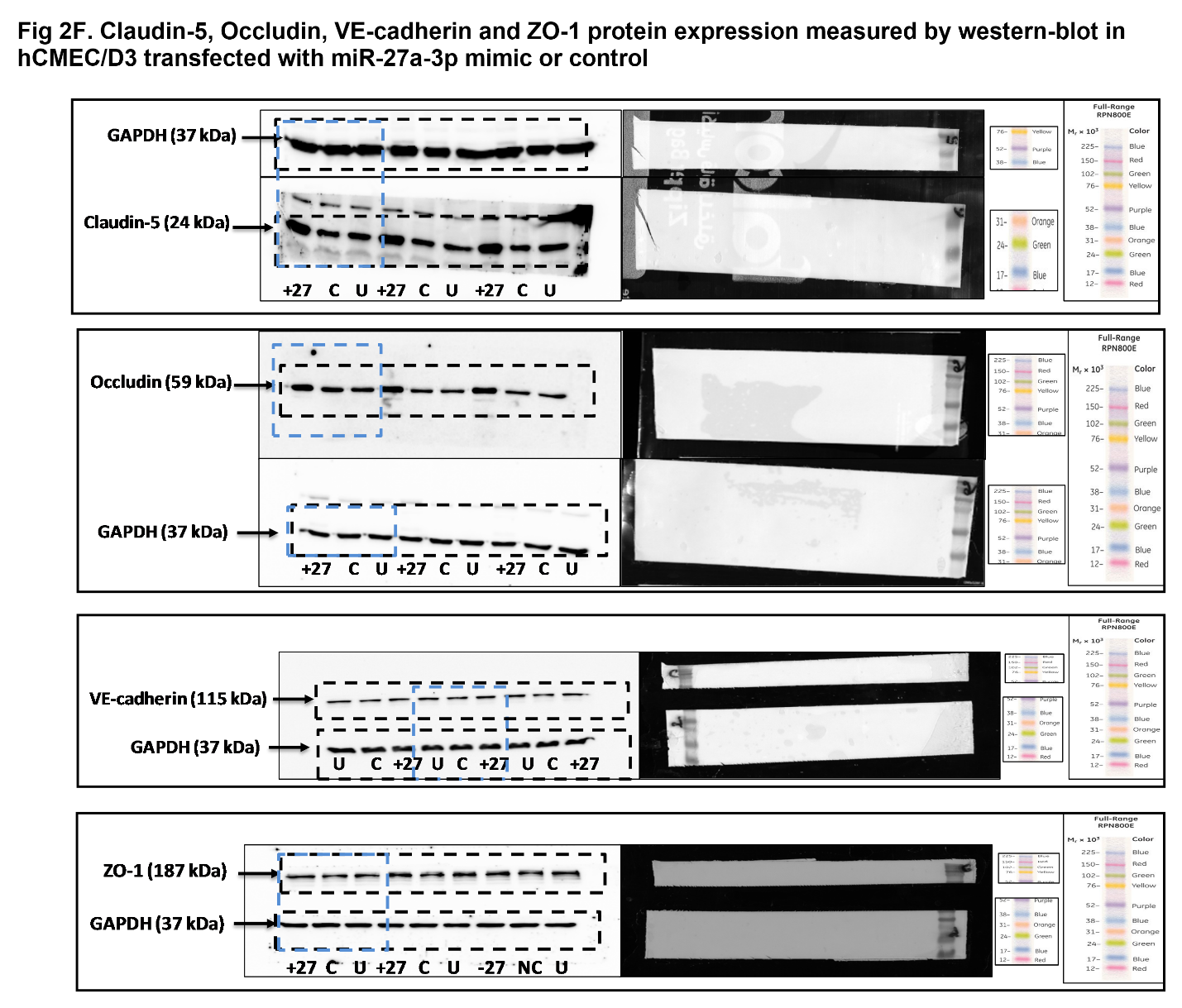

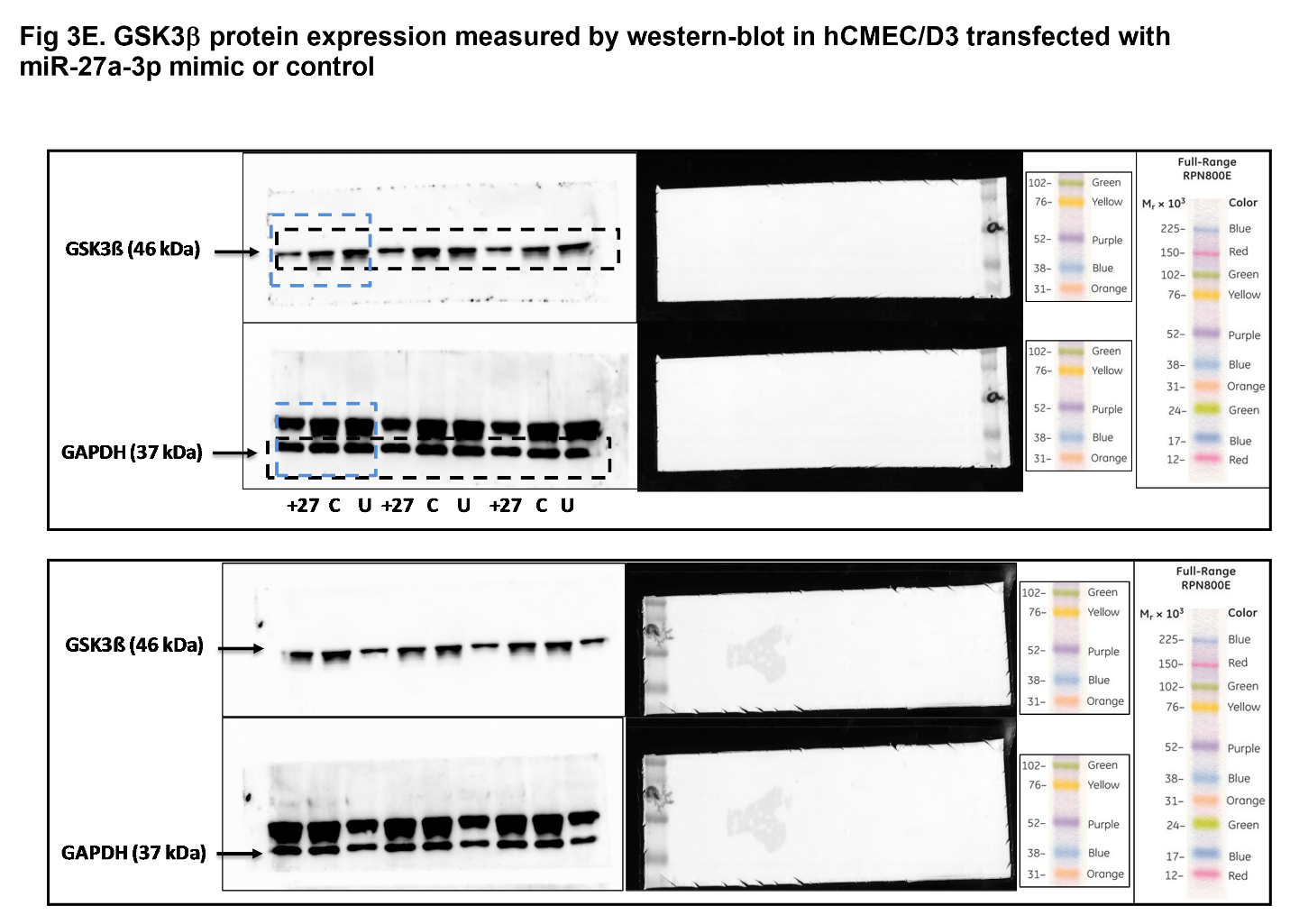

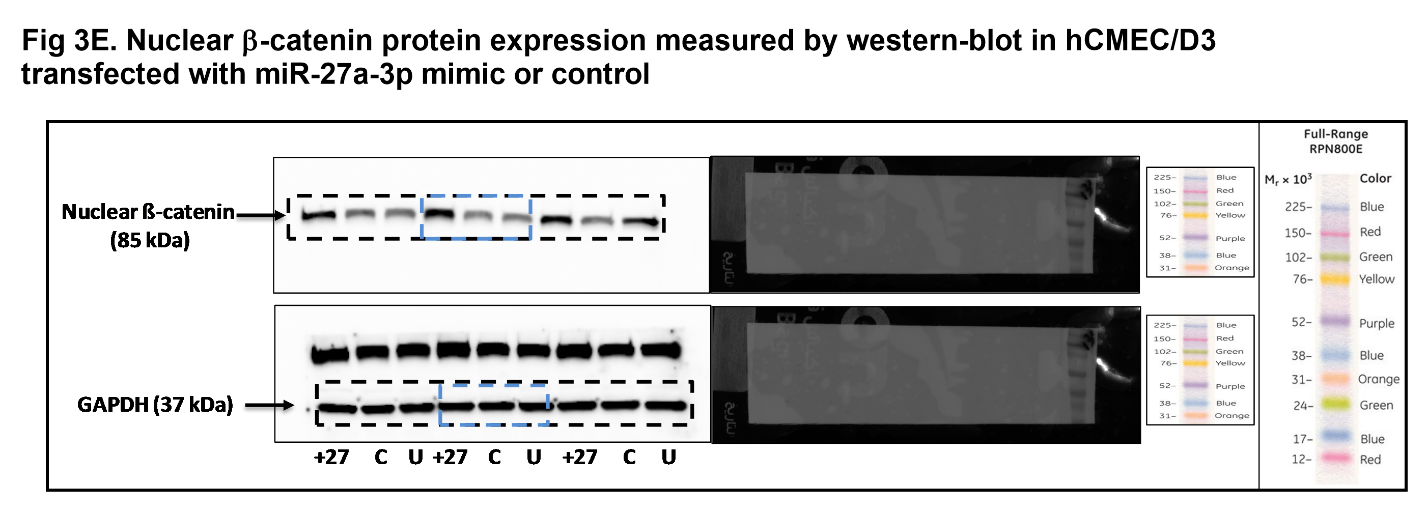

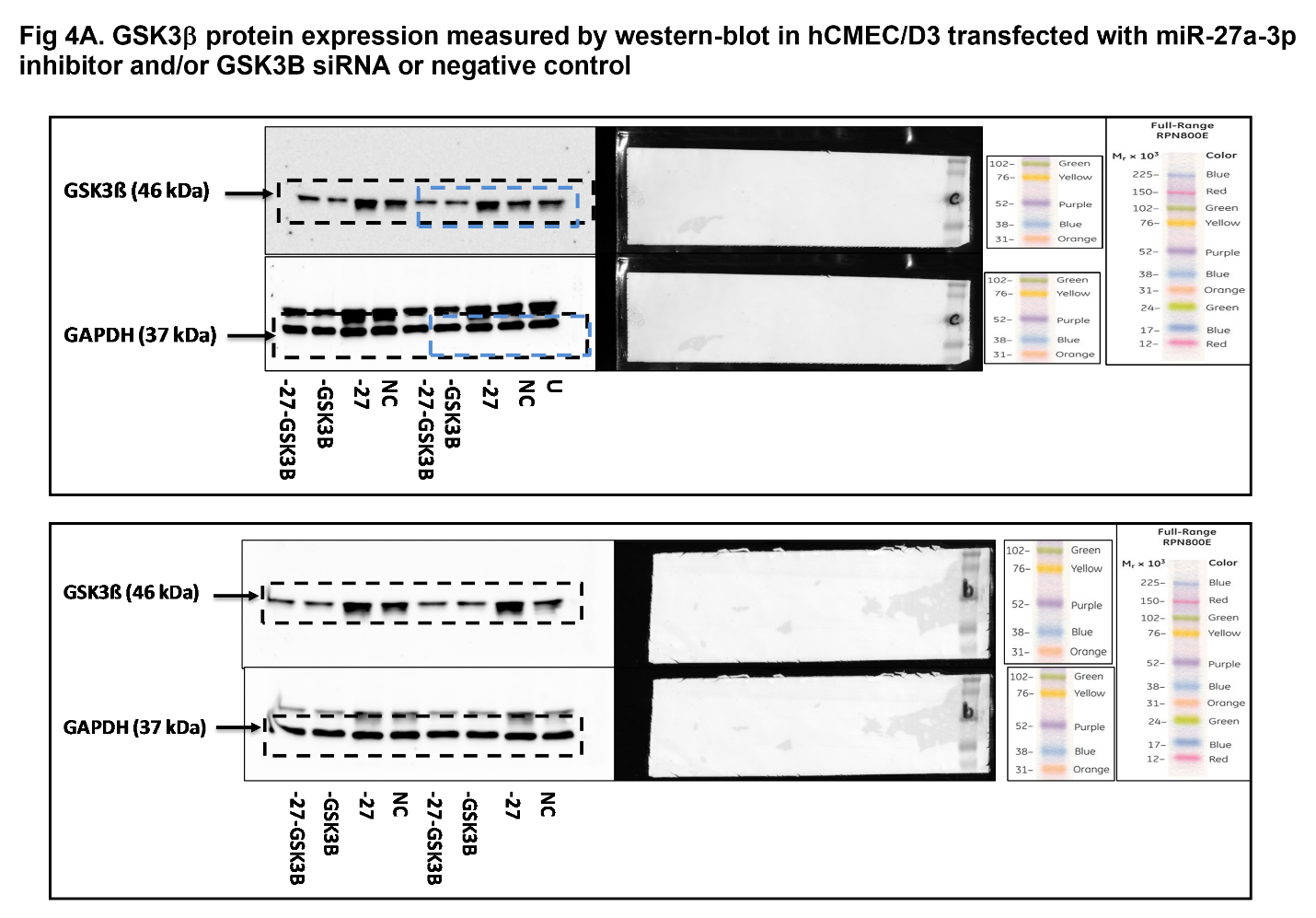

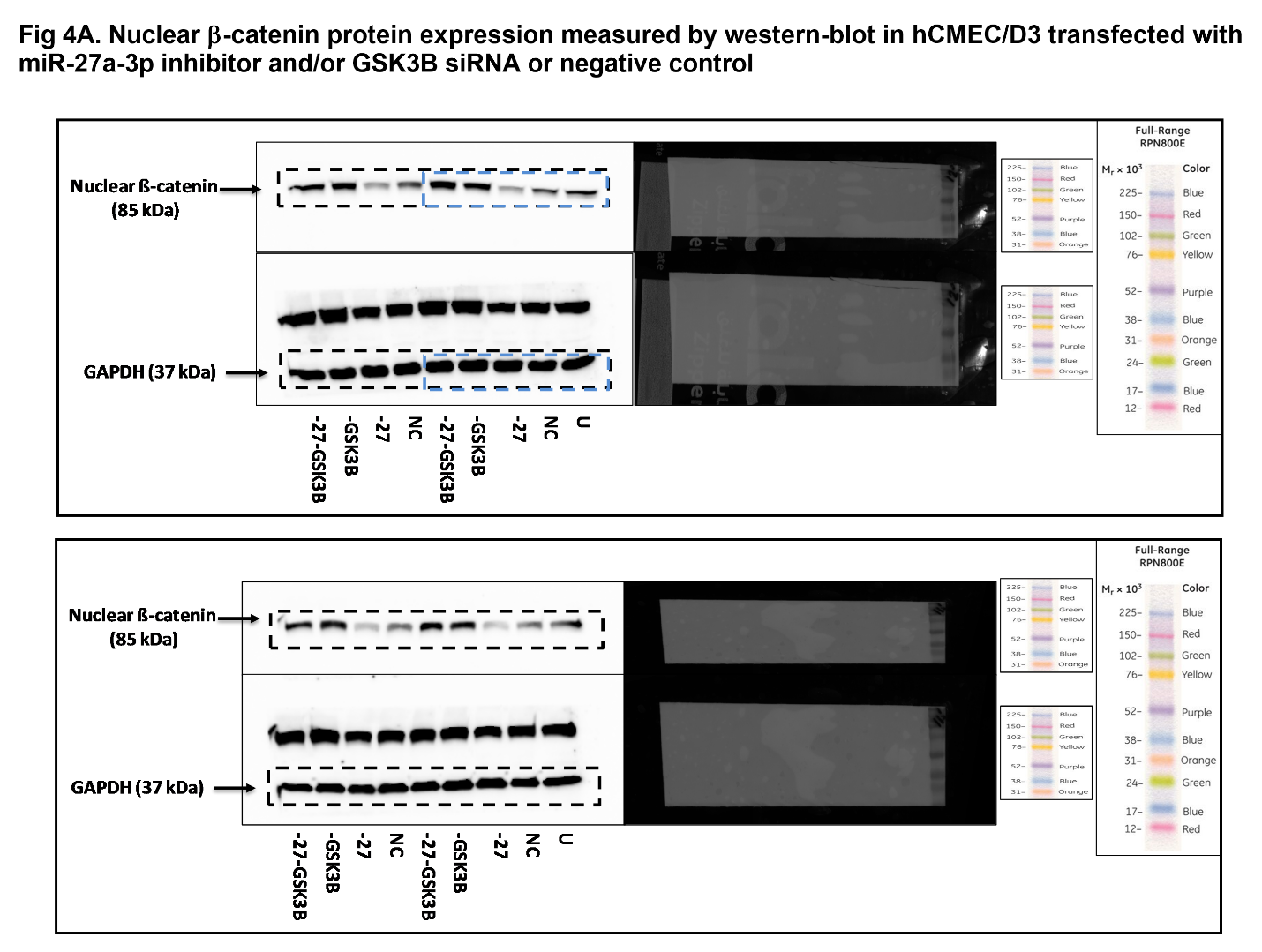

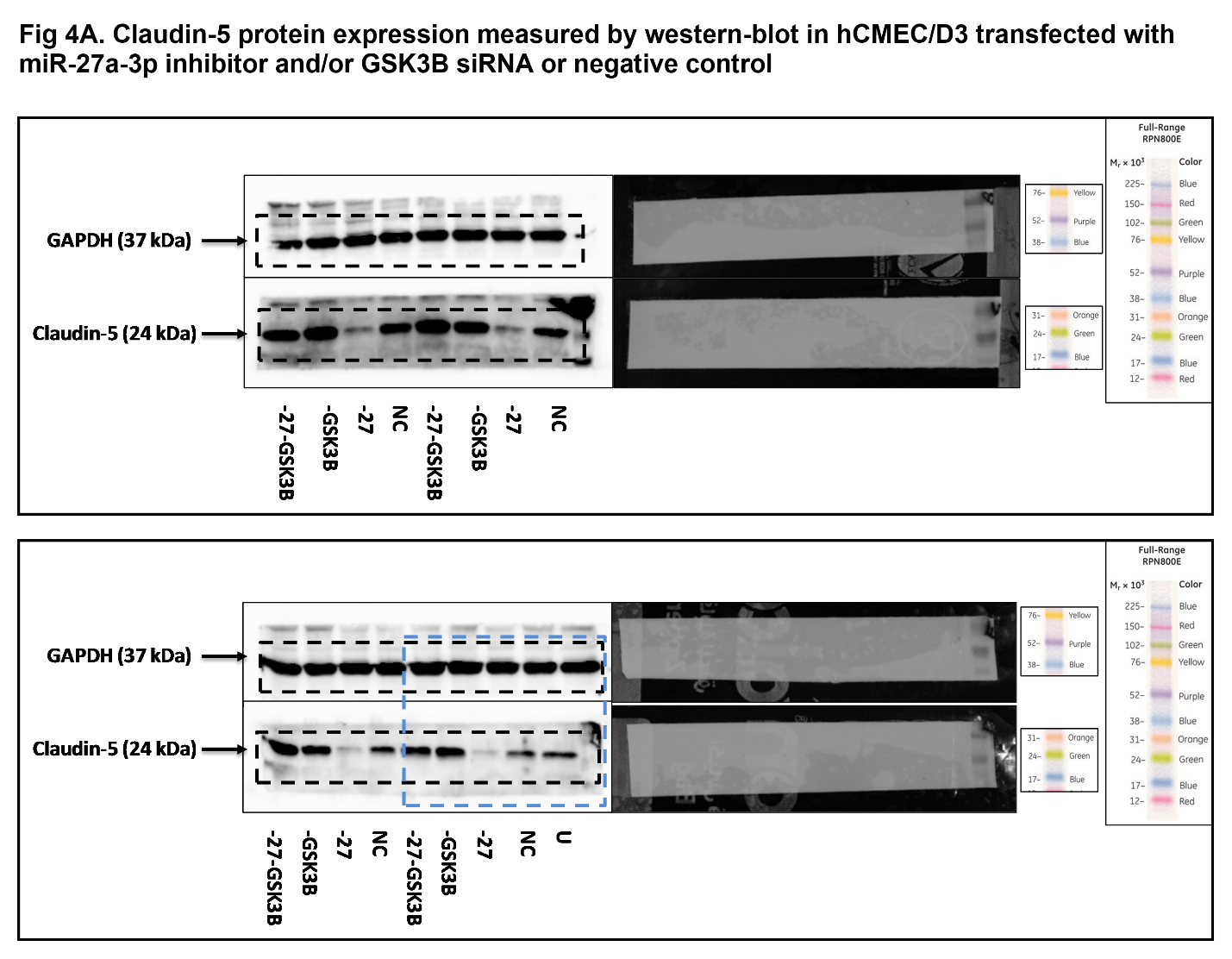

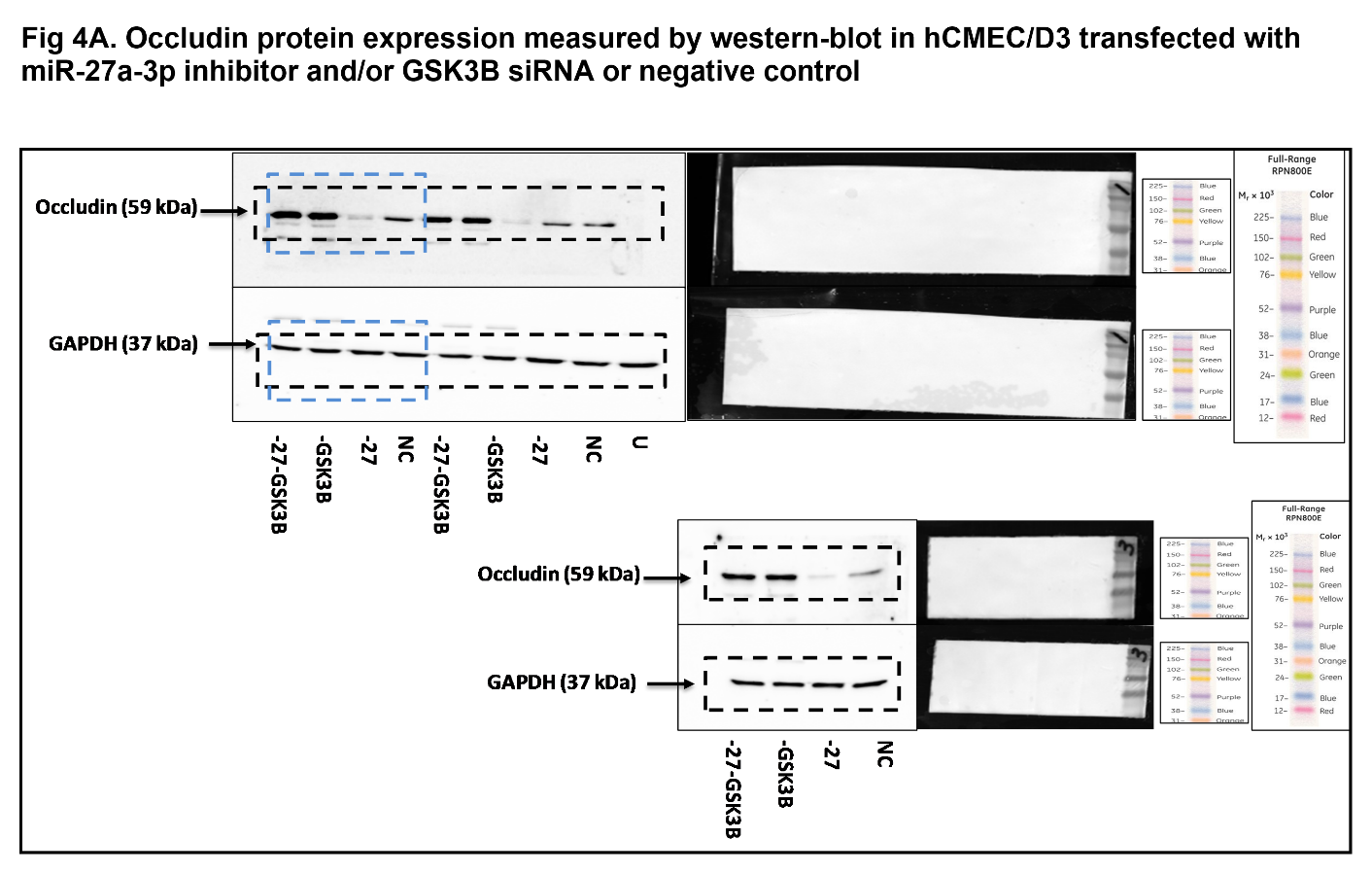

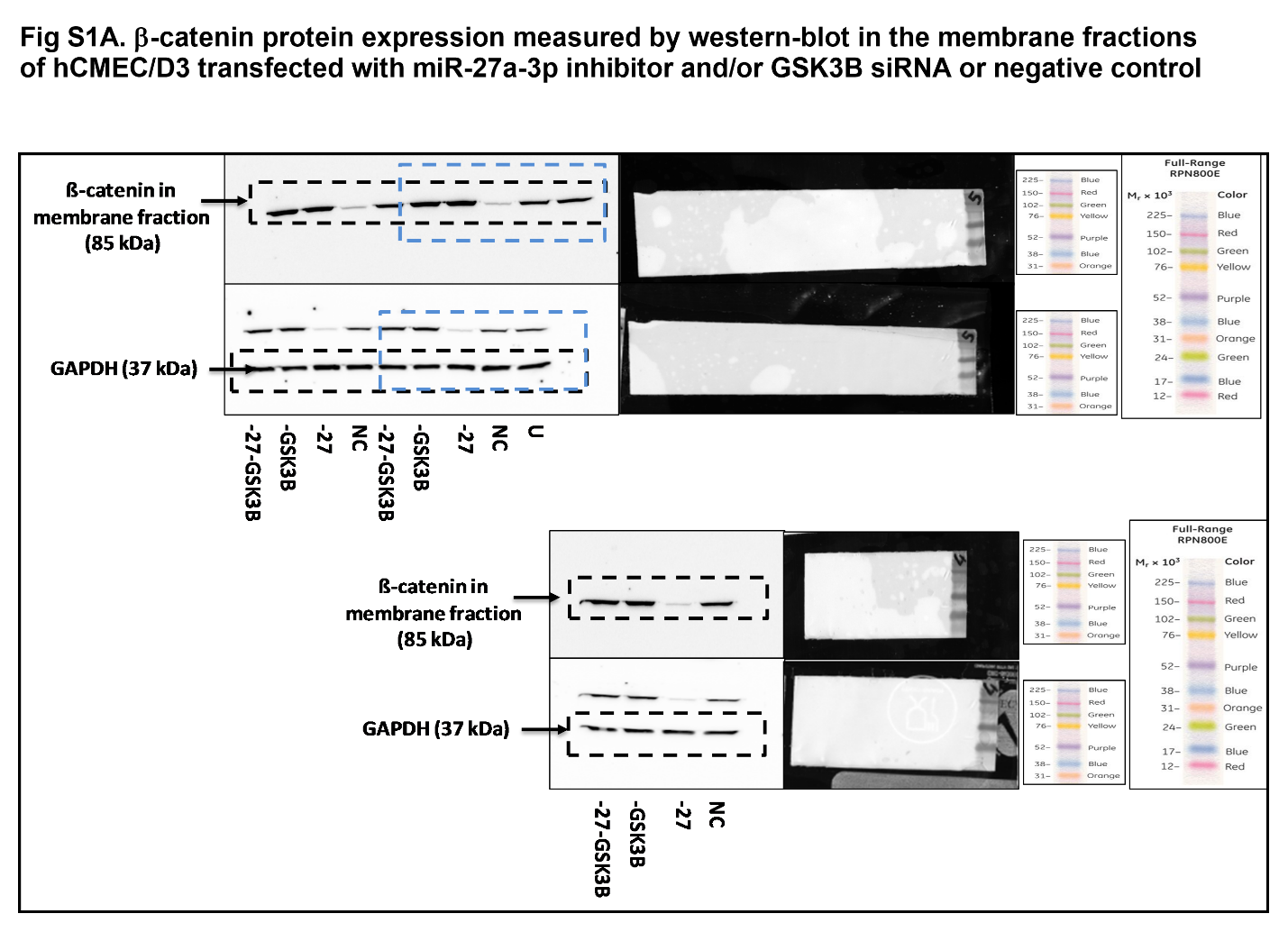

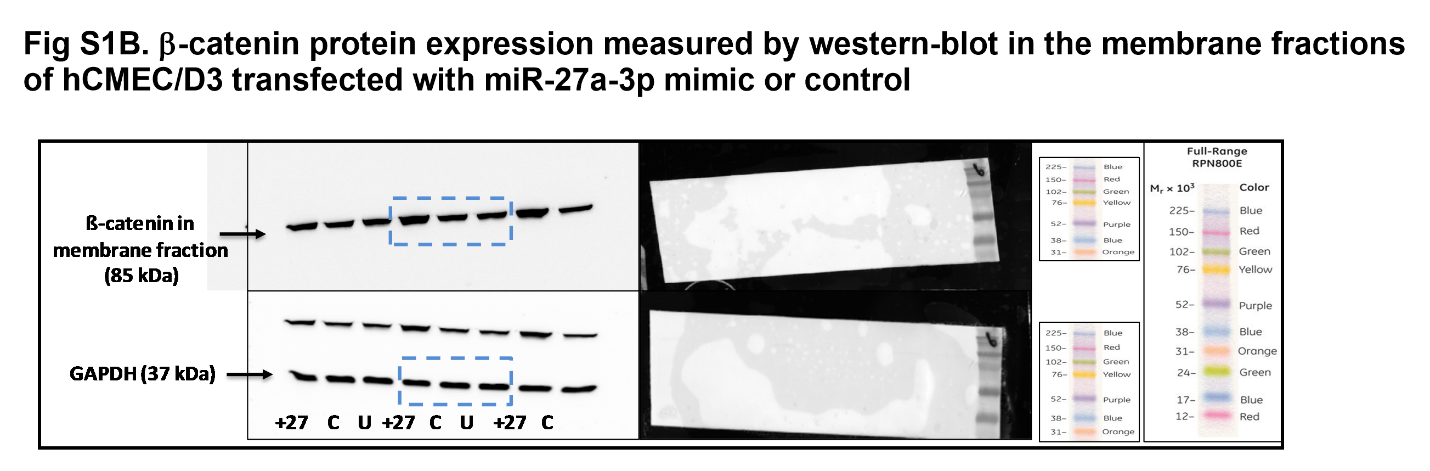
**
